# Transient tumor exposure induces persistent functional defects in memory CD8^+^ T cells

**DOI:** 10.1016/j.isci.2026.115556

**Published:** 2026-04-01

**Authors:** Daphné Laubreton, Margaux Prieux, Sophia Djebali, Maxence Dubois, Simon De Bernard, Olivier Gandrillon, Christophe Arpin, Jacqueline Marvel

**Affiliations:** 1 Centre International de Recherche en Infectiologie, Université de Lyon, INSERM U1111, CNRS UMR 5308, Ecole Normale Supérieure de Lyon, Université Claude Bernard Lyon 1, Lyon, France; 2 Laboratoire de Biologie et de Modélisation de la Cellule, Université de Lyon, ENS de Lyon, CNRS UMR 5239, INSERM U1210, Lyon, France; 3 AltraBio, Lyon, France; 4 Inria, Villeurbanne, France

**Keywords:** immunology, biological sciences, cancer

## Abstract

Memory CD8^+^ T cells generated during acute infections exhibit enhanced effector functions upon reactivation. However, persistent antigen exposure, such as in cancer, can impair their functionality. In this study, we compared memory CD8^+^ T cells generated following tumor rejection (Tum-CD8^+^) with those arising from an acute viral infection (Vir-CD8^+^). Using vaccinia virus and EL4 tumor models expressing the same antigen, we found that Tum-CD8^+^ cells displayed a distinct phenotype, including sustained expression of inhibitory receptors (PD-1, TIM-3), altered integrin expression, and reduced production of IFNγ and TNF. Despite retaining cytotoxic activity, their protective capacity was compromised, even after viral recall. Transcriptomic and functional analyses revealed that transient tumor exposure imprints a stable, exhaustion-like program on memory CD8^+^ T cells. These findings highlight how suboptimal priming conditions during tumor challenge durably shape memory T cell responses.

## Highlights

• Memory CD8^+^ T cells induced by a transient tumor challenge are impaired
• The exhaustion-like program imprinted by transient tumor challenge is irreversible
• This exhaustion-like program differs from that induced by chronic infections

## Graphical abstract

**Figure:**
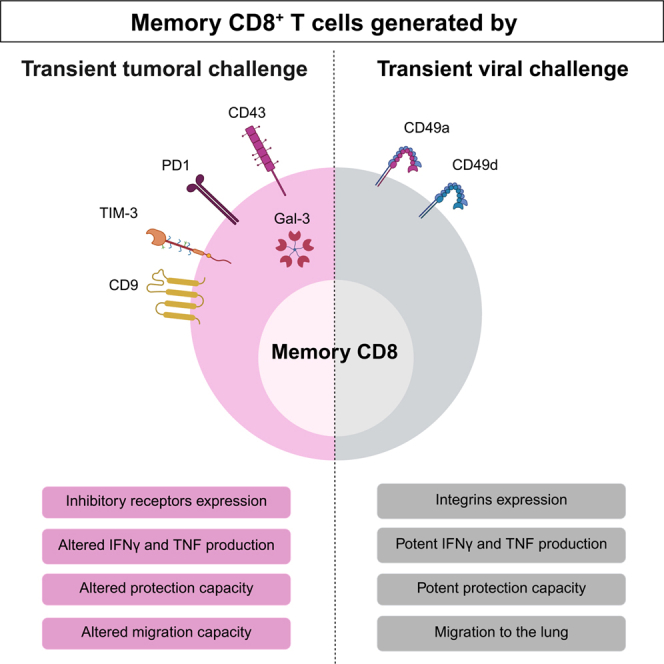

## Introduction

Following an acute infection, naive CD8^+^ T cells undergo extensive proliferation and differentiate into cytotoxic effector cells that control and clear the infection. After the resolution of the inflammation, most effector cells die, whereas a small fraction differentiates into memory cells. Unlike their naive counterparts, memory cells persist for extended periods^1^^,^^2^^,^^3^ and exhibit enhanced responsiveness upon antigen re-encounter.^4^^,^^5^ This state of hyper-responsiveness results from transcriptional and epigenetic modifications established upon activation and maintained thereafter.^6^^,^^7^^,^^8^ As a result, the functional properties of memory CD8^+^ T cells are modified, leading to immediate proliferation and rapid acquisition of effector functions, such as cytotoxicity or cytokine production, following recall. Moreover, they adopt new trafficking and homing potentials.^9^^,^^10^^,^^11^^,^^12^^,^^13^^,^^14^

In contrast, under conditions of persistent antigen exposure, such as chronic infections or cancer, memory CD8^+^ T cells enter a state of hyporesponsiveness, referred to as exhaustion.^15^ This state is characterized by impaired cellular functions, including reduced cytokine production, diminished proliferative capacity, and decreased cytotoxicity.^15^ Exhausted CD8^+^ T cells are characterized by a sustained expression of multiple cell-surface inhibitory receptors, including PD-1 or TIM-3^15^^,^^16^^,^^17^^,^^18^^,^^19^^,^^20^ as well as distinct transcriptomic and epigenetic programs,^18^^,^^21^^,^^22^^,^^23^^,^^24^ in both chronic infection or tumorigenesis. These cells originate from a precursor population (Tpex) that is characterized by high TCF1 expression.^25^^,^^26^^,^^27^^,^^28^

While exhausted CD8^+^ T cells arising in chronic infections and cancer share common exhaustion features, they also exhibit specific characteristics.^18^^,^^24^^,^^27^ In an antiviral environment, Tpex are generated in both acute and chronic contexts upon strong TCR priming and require continuous antigen exposure for their maintenance.^29^^,^^30^ However, in cancer, T cell priming is inefficient because tumor antigens are not presented in a proper inflammatory and co-stimulatory context, and that can lead to a state of anergy.^18^^,^^31^ Indeed, CD8^+^ T cells display altered cytokine production within the first few hours following *in vivo* activation by a tumor, a change that is associated with epigenetic modifications.^32^^,^^33^ These modifications include increased accessibility of the *Pdcd1* gene-enhancer, which regulates PD-1 expression.^32^^,^^33^ This state of unresponsiveness can lead to exhaustion if the tumor is not rejected, leading to the chronic stimulation of tumor antigen-specific T cells. However, the impact of a lack of effective costimulatory signals, in the absence of chronic antigen-exposure, on the responsiveness capacities of memory CD8^+^ T cells remains to be fully elucidated.

To answer that question, we conducted a detailed comparison of the functional properties of memory CD8^+^ T cells generated following tumor rejection, using acute viral stimulation as a gold standard for the generation of functional memory CD8^+^ T cells. Both the virus (vaccinia virus - VV) and the tumor (EL4 cells) expressed the NP68 epitope recognized by the F5 TCR transgene, and are able to induce the activation and proliferation of F5-transgenic CD8^+^ T cells. In these settings, the virus was cleared, or the tumor was rejected within 2 weeks of exposure. We showed that memory CD8^+^ T cells generated following the tumoral challenge displayed a distinct phenotype characterized by decreased integrins expression and increased expression of the inhibitory receptors PD-1 and TIM-3. This exhausted-like phenotype is associated with a reduced capacity for cytokine production and proliferation upon reactivation, leading to a defective protective function. Finally, we demonstrated that this phenotype was stable and could not be reversed, even after a subsequent viral challenge. Thus, our findings suggest that the suboptimal co-stimulation associated with the tumoral challenge is sufficient to establish a partially exhausted profile on memory CD8^+^ T cells.

## Results

### Tumor or virus-induced memory cells display a restricted number of phenotypic and transcriptional profile differences

**Figure 1:**
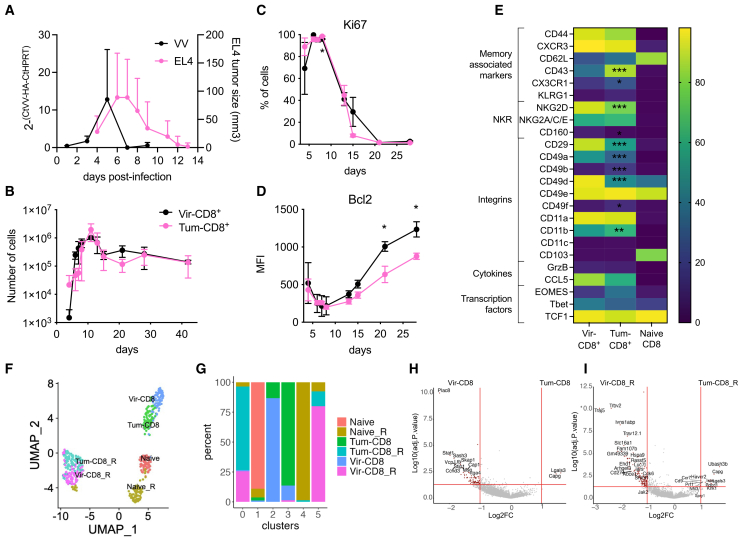
Phenotypic and transcriptional differences of memory CD8^+^ T cells generated after a viral or a tumoral challenge Naive F5 x CD45.1 cells (2.10^5^) were i.v. transferred in B6 mice 1-day prior immunization with VV-NP68 (i.n., 2.10^5^ pfu) or EL4-NP68 cells (s.c., 2,5.10^6^ cells). (A) Viral load was measured in the lung by qPCR, or tumor volume (mm^3^) was assessed by measuring its length, width, and thickness over time. (B) The number of Vir-CD8^+^ and Tum-CD8^+^ cells was determined over time in the blood by flow cytometry. (C and D) The expression of Ki67 (C) and Bcl2 (D) by Vir-CD8^+^ and Tum-CD8^+^ cells was measured over time in the blood. (E) The phenotype of Vir-CD8^+^ and Tum-CD8^+^ cells was analyzed 31 days after infection in the spleen, and the percentages of cells expressing each marker are represented as a heatmap. The statistical significance of differences was determined using a two-way ANOVA (C–E) (∗*p* < 0.05, ∗∗*p* < 0.01, and ∗∗∗*p* < 0.001). Data are represented as mean ± SD and are representative of 3 independent experiments (*n* = 5 to 10 mice per group). (F–I) 60 days after immunization, naive F5 and Vir-CD8^+^ and Tum-CD8^+^ cells were single-cell sorted, and stimulated with NP68 peptide (10 nM) for 2 h or left untreated. The transcriptome was analyzed by scRNAseq (*n* = 476 cells). (F) Clustering of cells projected on a UMAP colored by populations. (G) Proportion of sorted populations in each cluster. (H and I) Volcano plot of the differentially expressed genes between quiescent (H) or restimulated (I) Vir-CD8^+^ and Tum-CD8^+^.

To determine the impact of a transient tumoral challenge on the generation of CD8^+^ memory cells, we compared the phenotype of memory CD8^+^ T cells generated after a viral challenge with VV or a tumor challenge with EL4 cells. Maximal viral replication was detected 5 days post-challenge (dpc) while tumor size peaked between 6 and 7 dpc (Figure 1A). The virus and tumor were both eliminated from the challenge site within 7 and 12 dpc, respectively (Figure 1A). We first followed the number of F5 CD8^+^ T cells over time in the blood and showed that the number of circulating cells peaked at 11 dpc for both virus-challenged or tumor-challenged mice (Figure 1B). Furthermore, the number of CD8^+^ T cells recovered in the memory phase after a viral or a tumoral challenge was similar (Figure 1B). We then used Ki67 and Bcl2 labeling to compare the generation of early effector (EE, Ki67+ Bcl2-), late effectors (LE, Ki67- Bcl2-) and memory cells (Ki67- Bcl2+).^34^ The kinetics of EE generation, as well as the expression of Ki67 by CD8^+^ T cells, were similar between virus- and tumor-challenged mice (Figures 1C, S1A, and S1B). However, the number of LE was maintained for a longer period of 3–5 days, and there was a similar time delay in the appearance of memory cells following tumor challenge compared to viral challenge (Figures S1A and S1B). Furthermore, while both virus-induced (Vir-CD8^+^) and tumor-induced (Tum-CD8^+^) cells progressively reacquired the expression of Bcl2, its level remained lower at all time points in Tum-CD8^+^ cells (Figure 1D).

We then compared the phenotype of memory CD8^+^ T cells generated following both challenges. Compared to naive CD8^+^ T cells, Vir-CD8^+^ and Tum-CD8^+^ memory cells expressed surface markers associated with memory (Figures 1D and 1E). However, Tum-CD8^+^ memory cells exhibited reduced expression of NKG2D, as well as the integrins CD29, CD49a, and CD49d, which are involved in the migration of antigen-induced memory CD8^+^ T cells to inflamed tissues.^35^^,^^36^^,^^37^^,^^38^ Additionally, there was a lesser reduction in the expression of CD160 (Figures 1E and S1C). Moreover, Tum-CD8^+^ memory cells expressed higher levels of CX3CR1, CD49b, CD49f, CD11b, and, more importantly, CD43 compared to Vir-CD8^+^ memory cells (Figures 1E and S1C). Notably, the expression of CD43 is inversely correlated with recall response capacity.^39^

To fully characterize Vir-CD8^+^ and Tum-CD8^+^ memory cells, we analyzed the whole transcriptome at the single-cell level both *ex vivo* (Vir-CD8^+^, Tum-CD8^+^, Naive) and following a brief *in vitro* stimulation with NP68 peptide (Vir-CD8^+^_R, Tum-CD8^+^_R, Naive_R). Naive CD8^+^ T cells were included as a control. We performed a clustering analysis and projected the cells onto a UMAP (Figure 1F). We identified six clusters that were mainly defined by the experimental conditions, with memory cells generated by both challenges clustering together according to their activation status, and at a distance from naive cells (Figures 1G and S2A). This indicates that the memory cells generated following both challenges shared a significant portion of their memory gene expression signatures.

To unravel the differences in the transcriptomic profiles of Tum-CD8^+^ and Vir-CD8^+^ memory cells, we performed a differential expression analysis between these two cell types in quiescent (Figure 1H) or activated conditions (Figure 1I). Under quiescent conditions, Tum-CD8^+^ memory cells expressed higher levels of only two gene transcripts (*Lgals3* and *Capg*) but showed decreased levels of a larger number of genes*,* when compared to Vir-CD8^+^ memory cells (Figures 1H and S2B, Tables S1, and S2). These are associated with antiviral responses, such as *Stat1*, metabolic processes, such as *Vcp*, *Eno1,* or *Gimap7,* and cell adhesion, such as *Itga4*. Following *in vitro* activation, 12 genes were upregulated by Tum-CD8^+^ memory cells compared to Vir-CD8^+^ memory cells, including genes involved in effector responses (*Icos*, *Ccr7*, *Prf1*, *Cd9*), genes encoding inhibitory receptors (*Pdcd1*, *Havcr2*), as well as *Capg* and *Lgals3*. In contrast, Tum-CD8^+^ memory cells did not upregulate a large number of genes related to cell metabolism and nuclear transport, as Vir-CD8^+^ memory cells did (Figures 1I and S2B; Tables S1 and S2).

Globally, memory cells generated after a transient tumor challenge exhibit decreased Bcl2 and integrin expression, along with the upregulation of genes encoding inhibitory receptors.

### Tum-CD8^+^ memory cells express molecules associated with T cell exhaustion

**Figure 2:**
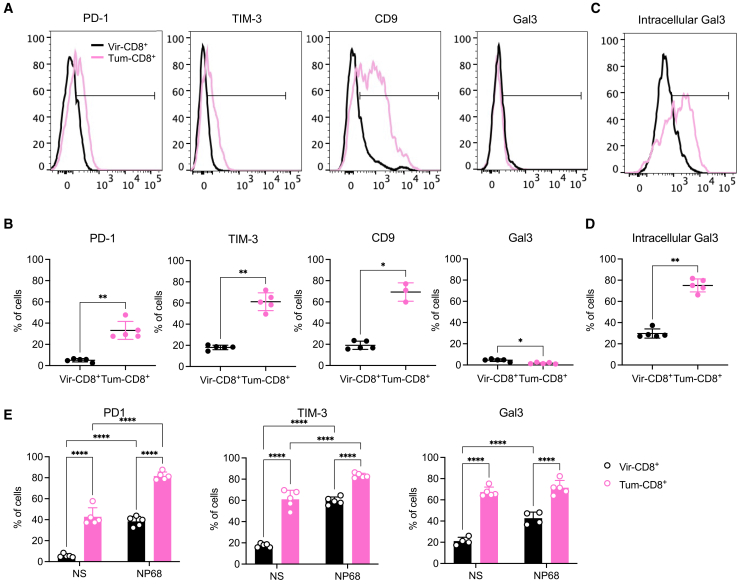
Tum-CD8^+^ memory cells express molecules associated with T cell exhaustion Naive F5 x CD45.1 cells (2.10^5^) were i.v. transferred in B6 mice 1-day prior immunization with VV-NP68 (i.n., 2.10^5^ pfu) or EL4-NP68 cells (s.c., 2,5.10^6^ cells). (A and B) The expression of PD-1, TIM-3, CD9, and Gal3 was measured at the surface of CD8^+^ memory cells at 30 dpi by flow cytometry. (C and D) The expression of Gal3 was measured intracellularly in memory CD8^+^ T cells at 30 dpi by flow cytometry. (E) At 30 dpi, splenocytes were stimulated with NP68 peptide (10 nM) for 4 h, and the expression of PD-1, TIM-3, or intracellular Gal3 by memory CD8^+^ T cells was measured by flow cytometry. The statistical significance of differences was determined using the Mann-Whitney test (B and D) or two-way ANOVA (E) (∗*p* < 0.05, ∗∗*p* < 0.01, and ∗∗∗∗*p* < 0.0001). Data are represented as mean ± SD (*n* = 5 mice per group) and are representative of 3 independent experiments.

We showed that quiescent or activated Tum-CD8^+^ memory cells overexpressed genes such as *Pdcd1* (PD-1), *Havcr2* (TIM-3), *Lgals3* (Gal3), and *Cd9* (CD9). Increased surface expression of all these markers except Gal3 was validated at the protein level for quiescent Tum-CD8^+^ memory cells, compared to Vir-CD8^+^ memory cells (Figures 2A and 2B).

As tumor size and exposure duration varied significantly among individual mice (Figures S3A and S3B), we next sought to determine whether the differential phenotype of Tum CD8^+^ cells was driven by antigen load or exposure duration, using tumor size at day 7 as a proxy for antigen burden (Figure S3C). No correlation was observed between tumor size and the expression of markers (PD-1, TIM-3, CD43, and CD49d) that distinguish Vir-CD8^+^ from Tum-CD8^+^ cells (Figure S3D). To further dissect this, we performed an additional experiment in which B6 × Ly5a hosts were immunized with EL4 tumor cells and received two populations of naive F5 T cells at different time points (CD45.2^+^ cells 1 day prior to tumor injection, and CD45.1^+^ cells 3 days later). This created a 3-day difference in antigen exposure between the two populations (Figure S3F). We selected this interval to minimize confounding effects from cytokines produced by activated T cells, as both adoptively transferred F5 CD8^+^ T cells and host T cells become activated 48–72 h post-tumor injection. At 34 dpi, we found no significant differences in the expression of PD-1, TIM-3, CD43, or CD49d between the two populations (Figure S3G). These results support the conclusion that the exhausted-like phenotype is driven by priming conditions rather than prolonged antigen exposure, consistent with our previous modeling results.^40^

Gal3 is a lectin with multiple functions in T cell biology.^41^ Specifically, it can alter cell functions and cytokine production by destabilizing the secretory synapse or modifying TCR sensitivity.^42^^,^^43^^,^^44^ Secreted Gal3 can bind to glycosylated proteins such as CD44 on the surface of T cells^41^ or be recruited intracellularly to the immunological synapse upon TCR stimulation.^45^^,^^46^ Thus, we determined the intracellular content of Gal3 and found that Tum-CD8^+^ memory cells contained higher levels of Gal3 than did Vir-CD8^+^ memory cells (Figures 2C and 2D).

The expression of these proteins was measured following NP68 peptide stimulation (Figure 2E). As observed at the gene level, the expression of both PD-1 and TIM-3 increased following activation but remained higher in Tum-CD8^+^ memory cells at all time points compared to Vir-CD8^+^ memory cells. However, intracellular Gal3 expression remained largely unchanged following *in vitro* stimulation, particularly in Tum-CD8^+^ memory cells. In summary, a transient tumoral challenge was sufficient to generate memory CD8^+^ T cells that express a restricted number of exhaustion markers and support the idea that tumor-specific priming, rather than chronic antigen exposure, drives this phenotype.

### Cytokine production, but not cytotoxicity, is altered in Tum-CD8^+^ memory cells

**Figure 3:**
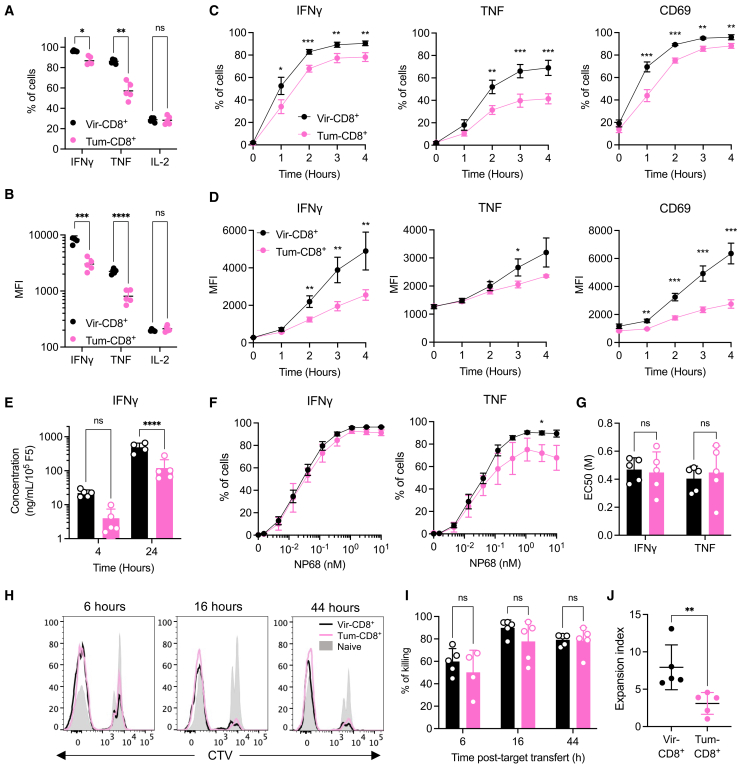
Tum-CD8^+^ memory cells display altered cytokine production but not cytotoxic capacities compared to Vir-CD8^+^ memory cells Naive F5 x CD45.1 cells (2.10^5^) were i.v. transferred in B6 mice 1-day prior immunization with VV-NP68 (i.n., 2.10^5^ pfu) or EL4-NP68 cells (s.c., 2,5.10^6^ cells). (A and B) At 30 dpi, F5 memory cells were restimulated with NP68 (10 nM) for 4h in the presence of GolgiStop. The production of IFNγ, TNF, and IL-2 was measured by flow cytometry and expressed in % of total F5 (A) or MFI within the cytokine+ cells (B). (C and D) At 30 dpi, F5 memory cells were restimulated with NP68 (10 nM) for 4h in the presence (cytokines) or absence (CD69) of GolgiStop. The production of IFNγ and TNF and the upregulation of CD69 were measured by flow cytometry over time and expressed in % (C) or MFI (D). (E) The production of IFNγ was measured in supernatant after 4 or 24h of stimulation. (F and G) At 30 dpi, F5 memory cells were restimulated with various doses of NP68 for 4h in the presence of GolgiStop, and the production of IFNγ and TNF was measured by flow cytometry (F). EC 50 was determined (G). (H and I) Splenocytes were incubated with NP68 (10 nM) or control medium for 2h and labeled with CTV or CFSE, respectively. A 1:1 ratio of NP68-loaded splenocytes: control splenocytes (2.10^6^ cells) was injected i.v. in Tum-CD8^+^ or Vir-CD8^+^ challenged mice at the memory stage. Representative histograms depicting control and CTV-labeled NP68-loaded splenocytes are shown (H). The percentage of NP68-loaded splenocytes killed was evaluated at 6-, 16-, or 44-h post-transfer (I). (J)Total CD8^+^ enriched from Tum-CD8^+^ or Vir-CD8^+^ challenged mice were labeled with CTV and stimulated with NP68-loaded DCs (1:1 ratio) for 4 days in the presence of IL-2. The expansion index of F5 cells was determined after 4 days. The statistical significance of differences was determined using 2-way ANOVA (∗*p* < 0.05, ∗∗*p* < 0.01, ∗∗∗*p* < 0.001, and ∗∗∗*p* < 0.0001). Data are represented as mean ± SD (*n* = 5 mice per group) and are representative of 3 independent (A–G) or 1 (H–J) experiment(s).

To define the functional consequences associated with the differential expression of Gal3 and inhibitory receptors in Tum-CD8^+^ memory cells, we analyzed their cytokine production and cytotoxic capacities. We first showed that following *in vitro* stimulation with NP68, both memory cell types exhibited comparable IL-2 production capacity (Figures 3A and 3B). In contrast, a smaller fraction of Tum-CD8^+^ memory cells produced IFNγ and TNF compared to Vir-CD8^+^ memory cells (Figure 3A). Furthermore, based on the MFI value, the level of intracellular cytokines was significantly higher in Vir-CD8^+^ memory cells (Figure 3B), suggesting that the cytokine production capacity of Tum-CD8^+^ memory cells was impaired. This was further confirmed by time-course analysis of cytokine production at the cellular level and in the supernatant. Indeed, IFNγ and TNF production were consistently lower in Tum-CD8^+^ compared to Vir-CD8^+^ memory cells (Figures 3C–3E). Furthermore, the levels of CD69 expressed at the cell surface remained lower in Tum-CD8^+^ memory cells than in Vir-CD8^+^ memory cells at all time-points (Figures 3C and 3D). Importantly, this decreased cytokine production was independent of antigen load or exposure duration (Figures S3E and S3I).

Given that the expression of Gal3 has been linked to reduced antigen sensitivity of CD8^+^ T cells following chronic infection,^43^ the antigen sensitivity of memory CD8^+^ T cells was assessed. Cells were stimulated with increasing doses of the NP68 peptide, and their cytokine production was measured after 4 h. Although the frequency of Tum-CD8^+^ memory cells producing IFNγ and TNF was lower at most peptide concentrations (Figure 3F), the NP68 concentration required to achieve 50% of the maximum cytokine production (effective concentration 50 [EC50]) was similar for Tum-CD8^+^ and Vir-CD8^+^ memory cells (Figures 3G and S4).

Next, we evaluated the cytotoxic function of the memory CD8^+^ T cells *in vivo*. Thirty days after the viral or tumoral challenge, mice were injected with a 1:1 ratio of NP68-loaded splenocytes to control splenocytes, and the killing of NP68-loaded splenocytes was assessed at various times post-transfer. A similar frequency of NP68-loaded cells was eliminated in both groups of mice at all-time points, indicating equivalent cytotoxic activity between Tum-CD8^+^ and Vir-CD8^+^ memory cells (Figures 3H and 3I).

Finally, we measured the proliferative capacity of memory CD8^+^ T cells in response to an *in vitro* stimulation with NP68-loaded bone marrow dendritic cells (BMDCs). After 4 days of culture, living CD8^+^ T cells were counted, and the expansion index was measured. The proliferative capacity of Tum-CD8^+^ memory cells was lower than that of Vir-CD8^+^ memory cells (Figure 3J). Overall, the memory cells generated following a transient tumoral challenge exhibited a reduced capacity for proliferation and production of TNF and IFNγ following secondary *in vitro* activation. However, their cytotoxic activity remained unaffected.

### A transient tumoral challenge is sufficient to impair the protective capacity of F5 memory cells

**Figure 4:**
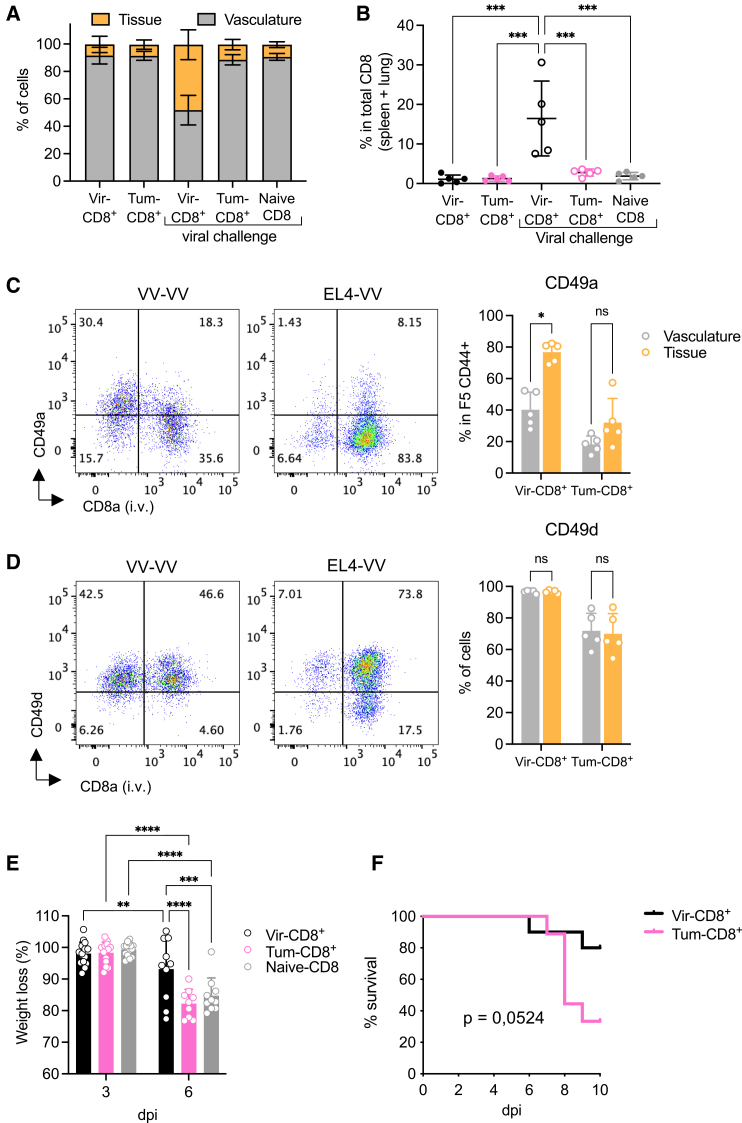
A transient tumoral challenge is sufficient to alter the protection capacity of F5 memory cells Naive F5 x CD45.1 cells (2.10^5^) were i.v. transferred in B6 mice 1-day prior immunization with VV-NP68 (i.n., 2.10^5^ pfu) or EL4-NP68 cells (s.c., 2,5.10^6^ cells). (A–D) At 30 dpi, Vir- or Tum-challenged mice were infected with VV-NP68 (2.10^5^ pfu). Six days after infection, mice received an i.v. injection of anti-CD8 antibody, and the proportion of cells within the tissue and the vasculature of the lung was determined (A). The proportion of memory CD8^+^ T cells in the lung tissue among all memory CD8^+^ T cells was determined (B). The expression of CD49a (C) and CD49d (D) was measured on memory CD8^+^ T cells within the lung tissue and vasculature. (E) At 30 dpi, Vir- or Tum-challenged mice were infected with Flu-NP68 (5.10^4^ TCID50), and the weight loss was followed for 6 days. (F) At 30 dpi, Vir-CD8^+^ and Tum-CD8^+^ memory cells were FACS-sorted and transferred into B6 host (1,2.10^5^ cells, i.v. route). One day after transfer, mice received a lethal dose of Flu-NP68 (2.10^6^ TCID 50), and survival was followed for 10 days. The statistical significance of differences was determined with 1-way (B) or 2-way (C–E) ANOVA test (∗*p* > 0.05, ∗∗*p* < 0.01, ∗∗∗*p* < 0.001, and ∗∗∗∗*p* < 0.0001), or log rank test (F). Data are represented as mean ± SD (*n* = 5 mice per group) and are representative of 2 (A–D, and G) or 1 (E and F) experiment(s).

Given that both virus and tumor cells were eliminated during the challenge, we next tested whether CD8^+^ memory cells were able to control a subsequent viral infection. Mice challenged with either virus or tumor were infected with VV-NP68 (intranasally) 30 days after the primary immunization. Six days later, the number and phenotype of F5 CD8^+^ cells were analyzed in the lung tissue and vasculature. The proportion of Tum-CD8^+^ memory cells within the lung tissue was lower than that of Vir-CD8^+^ memory cells following viral challenge (Figure 4A). To assess the capacity of memory CD8^+^ to be recruited to the lung, we determined the total number of F5 memory cells in the spleen and lung (Figure S5) and calculated the fraction of these cells recruited to the lung tissue for each mouse. Although the proportion of Tum-CD8^+^ and Vir-CD8^+^ memory cells within the lung tissue was similar under steady-state conditions, there was a strong recruitment of Vir-CD8^+^ memory cells, but not Tum-CD8^+^ memory cells or naive cells, to the lung tissue upon viral challenge (Figure 4B). This indicated that Vir-CD8^+^ memory cells have a better capacity to migrate to the infected site. This was correlated with the increased expression of CD49a on Vir-CD8^+^ T cells, but not Tum-CD8^+^ memory cells in the lung, an integrin involved in targeting cells to the lung^35^^,^^36^^,^^37^^,^^38^ (Figure 4C). However, the expression of CD49d did not differ between cells located in the vasculature and those in the tissue (Figure 4D).

We then wondered whether the reduced ability of Tum-CD8^+^ memory cells to enter the lung tissue would impact their protection capacity against heterologous infection with a Flu-virus carrying the NP68 epitope. We first addressed the capacity of Tum-CD8^+^ and Vir-CD8^+^ memory cells to protect mice from Flu-NP68 infection in a recall context. Virus- or tumor-challenged mice were infected with Flu-NP68 (i.n.) 30 days after the first immunization. Naive mice were used as controls. Similar to naive mice, the tumor-challenged mice lost nearly 20% of their initial body weight following Flu-NP68 infection, while virus-challenged mice lost only about 5% (Figure 4E).

Finally, to evaluate the role of Vir-CD8^+^ memory cells in the protection observed upon secondary viral challenge, we sorted Tum-CD8^+^ and Vir-CD8^+^ memory cells at 30 dpc and transferred them into naive B6 hosts. The day after, the mice received a lethal dose of Flu-NP68, and survival rates were monitored for 10 days (Figure 4G). Mice that received Tum-CD8^+^ memory cells exhibited a sharp decline in survival at 8 dpc, dropping to 30% survival by 10 dpc, whereas mice that received Vir-CD8^+^ memory cells maintained an 80% survival rate, indicating superior protection (Figure 4F). In conclusion, we demonstrated that while Tum-CD8^+^ memory cells were capable of rejecting the primary tumor and retaining certain effector functions, they proved ineffective against viral infection.

### Phenotype and cytokine production capacity of F5 memory cells is conserved after homologous or heterologous recall

**Figure 5:**
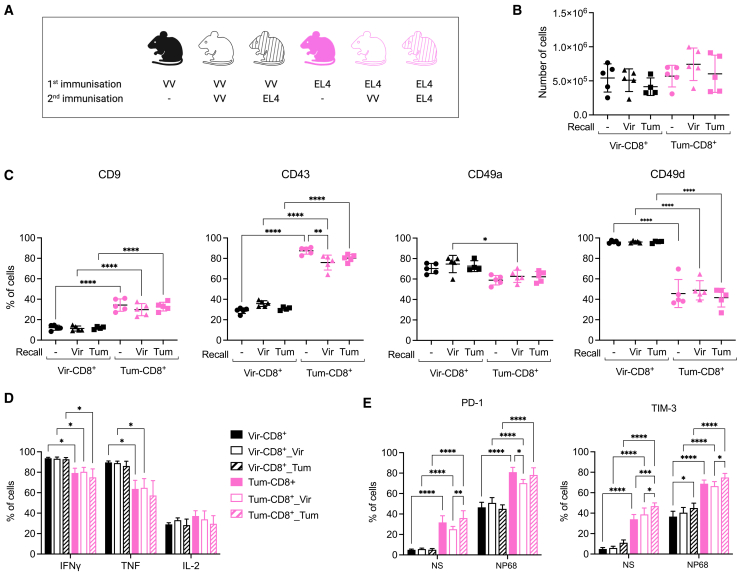
Phenotype and cytokine production capacity of F5 memory cells is conserved after homologous or heterologous recall (A) Naive F5 x CD45.1 cells (2.10^5^) were i.v. transferred in B6 mice 1-day prior immunization with VV-NP68 (i.n., 2.10^5^ pfu) or EL4-NP68 cells (s.c., 2,5.10^6^ cells). At 26 dpi, mice received a second immunization with VV-NP68 or EL4-NP68. (B) Thirty-one days post-recall, the number of F5 cells was measured in the spleen. (C) The expression of CD9, CD43, CD49a, and CD49d was measured on F5 memory cells 31 days after recall by flow cytometry. (D and E) Splenocytes were stimulated with NP68 (10 nM) for 4h in the presence (D) or absence (E) of GolgiStop. (D) The production of IFNγ, TFNα, and IL-2 was measured by flow cytometry. (E) The expression of PD-1 and TIM3 on F5 memory cells was determined in the spleen. The statistical significance of differences was determined with 1-way (B and C) or 2-way (D and E) ANOVA test (∗*p* > 0.05, ∗∗∗*p* < 0.001, and ∗∗∗∗*p* < 0.0001). Data are represented as mean ± SD (*n* = 5 mice per group) and are representative of 2 independent experiments.

Finally, we sought to evaluate the stability of the phenotype that was induced after a viral or a tumoral challenge. For this purpose, viral- or tumor-challenged mice received a second homologous or heterologous recall with either VV-NP68 or EL4-NP68 at 26 dpc (Figure 5A). Mice that did not receive a second immunization were used as controls. An equivalent number of F5 memory cells was recovered from mice in all groups at 31 days post-recall (Figure 5B). The expression of the main markers that differed between Tum-CD8^+^ and Vir-CD8^+^ memory cells after a primary challenge was determined in the spleen memory cells. The expression of CD43 was slightly increased in Vir-CD8^+^ memory cells following a viral recall and slightly reduced in Tum-CD8^+^ memory cells following a recall with either a virus or a tumor (Figure 5C). However, the phenotype of Tum-CD8^+^ and Vir-CD8^+^ memory cells was globally stable. Indeed, the increased expression of CD9 and CD43 in Tum-CD8^+^ memory cells was not reversed by viral challenge (Figure 5C). Furthermore, these cells did not show significantly increased expression of CD49a and CD49d, which was acquired by Vir-CD8^+^ memory cells following a primary viral challenge (Figure 5C). Similarly, Vir-CD8^+^ memory cells did not modify their primary phenotype following a tumor challenge (Figure 5C). The stability of the phenotype acquired during the primary challenge was also observed for cytokine production (Figure 5D) and for the expression of PD-1 and TIM-3 (Figure 5E), following peptide activation.

Overall, a transient viral or tumoral challenge is sufficient to establish a stable phenotype that shapes the functional quality of memory cells.

## Discussion

In this study, we investigated how a transient tumor challenge influences the generation of memory CD8^+^ T cells. While the overall formation of memory CD8^+^ T cells remained unaffected by tumor exposure, we found that even short-term tumor priming was sufficient to induce an exhausted-like phenotype. This was characterized by the elevated expression of inhibitory receptors, impaired cytokine production, and altered recall responses.

Quantitatively, we showed that tumoral and viral immunization led to the same number of CD8^+^ T memory cells. Similarly, it has been shown that CD8^+^ T cells exhibit comparable expansion during the first days following transfer in mice with established liver tumors or during acute infections.^33^ Qualitatively, single-cell RNA-seq analyses revealed similar transcriptomic profiles in Vir-CD8^+^ and Tum-CD8^+^ memory cells, and differential gene expression analysis highlighted differences in the level of gene expression rather than distinct gene expression patterns between Vir-CD8^+^ and Tum-CD8^+^ memory cells. However, the limited number of cells analyzed (approximately 80 per experimental condition) may not have been sufficient to capture subtle differences in the gene expression profiles.

However, we demonstrated that Tum-CD8^+^ memory cells were characterized by the increased expression of several markers implicated in the restriction of T cell functions, such as *Lgals3* (Gal3), *Cd9* (CD9), *Pdcd1* (PD-1), and *Havcr2* (TIM-3) genes, both at the RNA and protein levels. Some of these markers contribute to T-cell exhaustion. Specifically, PD-1 and TIM-3 are two inhibitory receptors whose sustained expression is a hallmark of exhausted T cells.^15^ PD-1 restricts the expression of cytokines, such as IFNγ or TNF, in an IRF4-dependent manner^47^ while TIM-3 alters IL-2 production and limits the upregulation of CD69.^48^ Gal3 also plays a role in inducing the exhaustion program, possibly through excessive activation of NFAT signaling.^44^ Notably, Gal3-deficient CD8^+^ T cells displayed increased IFNγ and IL-2 production as well as enhanced proliferation compared to WT CD8^+^ T cells.^44^^,^^46^ Additionally, Gal3 and CD9 are negative regulators of the immunological synapse.^45^^,^^46^^,^^49^ Gal3 can co-localize with Zap70 and restrict TCR signaling,^46^ whereas CD9 competes with ICAM-1 for LFA-1 adhesion.^49^

We demonstrated that the exhausted-like phenotype exhibited by Tum-CD8^+^ memory cells is associated with altered functionality. Specifically, these cells showed a reduced capacity to upregulate CD69 and produce IFNγ and TNF cytokines in response to *in vitro* TCR stimulation. Furthermore, Tum-CD8^+^ T cells displayed reduced expansion capacity in response to *in vitro* stimulation compared to those generated following viral infection. However, Tum-CD8^+^ memory cells retained their capacity to kill target cells *in vivo* and to produce IL-2 following peptide restimulation*.* This finding contrasts with studies showing that IL-2 production is the first function lost during the exhaustion process in the context of chronic infections with LCMV.^15^^,^^50^

In addition to their expression of inhibitory receptors, Tum-CD8^+^ memory cells were characterized by the increased expression of the activation-associated glycoform of CD43, whose expression is associated with a lower recall capacity.^39^ In agreement with these findings, we showed that the capacity of Tum-CD8^+^ memory cells to protect against a Flu-infection was impaired as compared to Vir-CD8^+^ memory cells. Furthermore, Tum-CD8^+^ memory cells showed decreased expression of the integrins CD49a and CD49d. CD49a upregulation by CD8^+^ T cells is driven by inflammatory cytokines such as TGFβ, IL-6, and IL-12.^51^ Thus, the lower CD49a expression observed in Tum-CD8^+^ memory cells compared to Vir-CD8^+^ memory cells likely reflects the distinct inflammatory environment present in the tumor context. Notably, both CD49a and CD49d integrins are crucial for lung tissue homing.^35^^,^^36^^,^^37^^,^^38^ Consistent with their phenotype, we demonstrated that Tum-CD8^+^ memory cells were significantly less efficient than Vir-CD8^+^ memory cells in infiltrating lung tissue following recall with VV-NP68.

Exhausted CD8^+^ T cells arising in chronic infections and cancer exhibit environment-specific characteristics.^18^^,^^24^^,^^27^ For instance, NFAT5 regulates T cell exhaustion specifically in the hyperosmotic tumor microenvironment but not in chronic viral infections.^52^ Here, we demonstrate that tumor priming alone, without ongoing tumor burden, is sufficient to disrupt memory CD8^+^ T cell functionality and induce an exhausted-like state. Furthermore, we showed that CD8^+^ T cells activated in a tumoral context exhibited defects in inflammation-associated gene expression, such as *Stat1*, compared to those generated following viral infection. These findings highlight the crucial role of inflammatory signals in the generation of functional memory CD8^+^ T cells.

The exhausted T cell program that develops during chronic infection is fixed and unresponsive to PD-1 blockade.^53^ Our data extend this concept by showing that even a transient tumor challenge induces a partially exhausted phenotype that is similarly persistent and irreversible. Indeed, Tum-CD8^+^ memory cells maintained their unique phenotypic characteristics and impaired cytokine production, even after recall with VV-NP68 in both homologous and heterologous settings. This aligns with recent findings showing that a tumoral challenge induces epigenetic modifications at the *Pdcd1* locus within the first hours following CD8^+^ T cell activation.^33^ Further studies are needed to determine the epigenetic changes associated with a transient tumoral challenge. Similarly, Vir-CD8^+^ memory cells maintained their phenotype and function upon both homologous and heterologous recall, indicating that the positive traits established in virus-induced memory cells were stable and could not be reversed by the suboptimal stimulation associated with the tumor.

Chronic infections are classical models for studying T cell exhaustion, where persistent antigen exposure drives progressive functional decline, epigenetic remodeling, and sustained expression of inhibitory receptors.^15^ In our model of resolved tumor challenge, where antigen exposure does not persist, Tum-CD8^+^ memory cells exhibited hallmark features of exhaustion, including elevated PD-1 and TIM-3 expression, impaired IFNγ/TNF production, and reduced protection capacity. However, unlike terminally exhausted T cells in chronic settings, which often lose cytotoxic function prior to cytokine production,^50^^,^^54^ Tum-CD8^+^ cells retained their cytotoxic activity. This divergence suggests that suboptimal priming during transient tumor exposure may imprint a partial exhaustion program, distinct from the global hyporesponsiveness induced by chronic antigen stimulation. Future studies comparing the epigenetic and transcriptional landscapes of Tum-CD8^+^ cells with those from chronic tumor or infection models will be critical to dissect the mechanisms underlying these distinct exhaustion states.

Overall, our study emphasizes the importance of investigating the mechanisms driving CD8^+^ T cell dysfunction associated with suboptimal stimulation.

### Limitations of the study

While this study demonstrates that transient tumor exposure imprints a stable exhaustion-like program on memory CD8^+^ T cells, several caveats should be considered. First, we used a single tumor model (EL4), and comparing multiple tumor types with varying immunogenicity or microenvironments could provide further insights into the generalizability of the observed exhaustion-like program. Second, the single-cell RNA sequencing analysis, though informative, was limited by the number of cells analyzed (∼80 per condition), which may have restricted the detection of subtle transcriptional differences between Tum-CD8^+^ and Vir-CD8^+^ memory cells. Finally, while we demonstrate the stability of the exhaustion-like phenotype imprinted following transient tumoral challenge, the epigenetic mechanisms underlying its irreversibility remain to be fully characterized.

## Resource availability

### Lead contact

Further information and requests for resources should be directed to and will be fulfilled by the lead contact, Jacqueline Marvel (jacqueline.marvel@inserm.fr).

### Materials availability

This study did not generate new unique reagents.

### Data and code availability

• The sequencing data generated in this study are available at GEO NCBI under the accession number GEO: GSE283102.
• This paper does not report original code. Code used to generate figures is available upon reasonable request from the lead contact.
• Any additional information required to reanalyze the data reported in this paper is available from the lead contact upon request.

## Acknowledgments

We acknowledge the contributions of SFR BioSciences (UAR3444/CNRS, US8/Inserm, 10.13039/501100018692École Normale Supérieure de Lyon, 10.13039/501100011074Université de Lyon) and of the CELPHEDIA infrastructure (http://www.celphedia.eu/), especially the center AniRA in Lyon (AniRA-Cytométrie and AniRA-PBES facilities) and Yann Leverrier. We acknowledge Severine Valsesia and Mélanie Wencker for their help with the RNA library preparation. We acknowledge Laurent Modolo for his assistance in the analysis of single-cell RNA-Seq and Omran Allatif for statistical analysis. This work was supported by 10.13039/501100001677Inserm, 10.13039/501100004794CNRS, 10.13039/501100011074Université de Lyon, 10.13039/501100018692ENS de Lyon, 10.13039/501100010115Région Auvergne-Rhône-Alpes (Ingerence pack ambition), and ANR (MEMOIRE ANR-18-CE45-0001). Margaux Prieux has a région Auvergne-Rhône-Alpes Ph.D. fellowship. Part of some figures were created with BioRender.com.

## Author contributions

Conceptualization, C.A., D.L, M.P., and J.M.; data curation, S.D-B.; formal analysis, D.L. and M.P.; funding acquisition, J.M. and O.G.; investigation, C.A., D.L., M.D., M.P.,S.D., and S.D-B.; methodology, C.A., D.L., M.P., and J.M.; project administration, J.M.; resources, D.L, M.P., and J.M.; supervision, C.A. and J.M.; validation: D.L.; visualization: D.L. and J.M.; writing – original draft: D.L. and J.M; Writing – review and editing: C.A., M.P., O.G., and S.D.

## Declaration of interests

The authors declare no competing interests.

## Declaration of generative AI and AI-assisted technologies in the writing process

During the preparation of this work, the author(s) used ChatGPT (OpenAI) and Le Chat (Mistral AI) in order to correct the English grammar of the manuscript. After using these tools, the authors reviewed and edited the content as needed and take full responsibility for the content of the publication.

## STAR★Methods

### Key resources table

**Table:** 

REAGENT or RESOURCE	SOURCE	IDENTIFIER
**Antibodies**		

BUV395 anti-mouse CD8a (clone 53–6.7)	Waters^TM^ Biosciences	Cat#563786; RRID: AB_2732919
BUV737 anti-mou se CD45.1 (clone A20)	Waters^TM^ Biosciences	Cat#612811; RRID: AB_2738850
BV605 anti-mouse CD49a (clone Ha31/8)	Waters^TM^ Biosciences	Cat#740519; RRID: AB_2740235
FITC anti-mouse IFNg (clone XMG1.2)	Waters^TM^ Biosciences	Cat#562019; RRID: AB_395375
PE anti-mouse CD49a (clone Ha31/8)	Waters^TM^ Biosciences	Cat#562115; RRID: AB_11153117
PE-Cyanine7 anti-mouse CD69	Waters^TM^ Biosciences	Cat#561930; RRID: AB_394508
APC anti-mouse CD43 Activation-Associated Glycoform	Biolegend	Cat#121213; RRID: AB_528806
APC anti-mouse IL-2	Biolegend	Cat#503809; RRID: AB_315303
BV421 anti-mouse/human Mac-2 (Galectin-3)	Biolegend	Cat#125416; RRID: AB_2566686
BV605 anti-mouse CD45.1	Biolegend	Cat#110737; RRID: AB_11204076
BV605 anti-mouse/human CD44	Biolegend	Cat#103047; RRID: AB_2562451
BV650 anti-mouse TNFa	Biolegend	Cat#506333; RRID: AB_2562450
BV711 anti-mouse CD8a	Biolegend	Cat#100748; RRID: AB_2562100
FITC anti-rat/mouse-Bcl-2	Biolegend	Cat#633504; RRID: AB_2028394
PE anti-mouse CD43 Activation-Associated Glycoform	Biolegend	Cat#121208; RRID: AB_493388
PE-Cyanine7 anti-mouse CD9	Biolegend	Cat#124815; RRID: AB_2783074
PE/Dazzle 594 anti-mouse CD279 (PD-1)	Biolegend	Cat#109116; RRID: AB_2566548
VioBlue anti-mouse CD44	Miltenyi Biotec	Cat#130-102-443; RRID: AB_2658185
PE anti-mouse TIM-3	R&D Systems	Cat#FAB1529P; RRID: AB_2114281
PE-Cyanine7 anti-mouse CD8a	Thermo Fischer Scientific	Cat#25-0081-82; RRID: AB_469584
PerCP-eFluor 710 anti-mouse CD49d	Thermo Fischer Scientific	Cat#46-0492-80; RRID: AB_11150051
PerCP-eFluor 710 anti-mouse Ki67	Thermo Fischer Scientific	Cat#46-5698-82; RRID: AB_11040981

**Bacterial and virus strains**		

VV-NP68	Dr. D.Y.-L. Teoh (Human Immunology Unit, Institute of Molecular Medicine, Oxford, U.K.)	Modified from the Western Reserve strain
Flu-NP68	Dr. Olivier Ferraris and Dr. Michelle Ottmann in Pr. Bruno Lina’s laboratory at the Université de Lyon	Modified from the A/WSN/33 H1N1 strain

**Chemicals, peptides, and recombinant proteins**		

DMEM	Thermo Fischer Scientific	Cat#61965-026
Sodium pyruvate (100 mM)	Thermo Fischer Scientific	Cat#11360-039rc
HEPES (1M)	Thermo Fischer Scientific	Cat#15630-056
Gentamicin (50 mg/mL)	Thermo Fischer Scientific	Cat#15750-037
Beta-mercaptoethanol	Thermo Fischer Scientific	Cat#31350-010
DPBS	Thermo Fischer Scientific	Cat#14190-094
FBS	Biowest	Cat#S1810-500 (Lot#S13439S1810)
efluor780-coupled Fixable Viability Dye	Invitrogen	Cat#65-0865-18
CellTrace CFSE Cell Proliferation Kit	Invitrogen	Cat#C34554
CellTrace Violet Cell Proliferation Kit	Invitrogen	Cat#C34557
GolgiStop^TM^	Waters^TM^ Biosciences	Cat#554724
Flow-count fluorospheres	Beckman Coulter	Cat#7547053
NP68 (ASNENMDAM)	Proteogenix	N/A
CpG ODN 1826	InvivoGen	tlrl-1826
human Flt3l	Amgen	N/A
RNaseOut	Thermo Fischer Scientific	Cat#10777019
Random primers	Thermo Fischer Scientific	Cat#N8080127
dNTPs	Thermo Fischer Scientific	Cat#R0192
Superscript II	Thermo Fischer Scientific	Cat#18064014
DTT	Thermo Fischer Scientific	Cat#18064014
MgCl2	Sigma	Cat# M1028-10X1ML
KAPA Hifi HotStart Ready Mix	KAPA Biosystem	Cat#KK2602
Agencourt AMPure XP	Beckman	Cat# A63880
Nextera XT DNA sample preparation kit	Illumina	Cat#FC-131-1096
Nextera XT 96-index kit	Illumina	Cat#FC-131-1002

**Critical commercial assays**		

IFN-g ELISA MAX^TM^ standard Set mouse kit	Biolegend	Cat#130-104-075; RRID:AB_2893366
Lung Dissociation kit	Miltenyi Biotech	Cat# 130-095-927; RRID:SCR_020286
CD8a+ T cell isolation kit	Miltenyi Biotec	Cat#130-104-075
Foxp3/Transcription Factor Staining Buffer Set kit	eBioscience	Cat#00-5523-00
Cytofix/Cytoperm	Waters^TM^ Biosciences	Cat#554714; RRID: AB_2869008
Flow-count fluorospheres	Beckman Coulter	Cat#7547053
NucleoSpin Tissue kit	Macherey Nagel	740952.50
Platinum® SYBR® Green qPCR SuperMix-UDG with ROX	Thermo Fischer Scientific	11744100
DNA high sensitivity D1000 ScreenTape	Agilent	Cat#5067-5584

**Deposited data**		

Raw and analyzed data	This paper	GEO: GSE283102

**Experimental models: Cell lines**		

EL4-NP68	Dr. T.N.M. Schumacher	Modified from EL4 lymphoma cell line (ATCC “TIB-39”)

**Experimental models: Organisms/strains**		

C57Bl6/J	Charles River	MGI:3028467
F5 x CD45.1 (B6-^Ptprcem(K302E)Jmar^/J-Tg(CD2-TcraF5,CD2-TcrbF5)1Kio/Jmar)	This paper	RRID:IMSR_EM:15572

**Oligonucleotides**		

VV-HA-Forward (CATCATCTGGAATT GTCACTACTAAA)	Sigma	N/A
VV-HA-Reverse (ACGGCCGACATAT AATTAATGC)	Sigma	N/A
HPRT-Forward (AAAGACTTGCTCGAGATG)	Sigma	N/A
HPRT-Reverse (TAATGTAAT-CCAGCAGGTC)	Sigma	N/A

**Software and algorithms**		

BD FACSDiva (v8.0) software	Waters^TM^ Biosciences	N/A
Flowjo (v10.8.0)	Waters^TM^ Biosciences	RRID:SCR_008520
Prism (v10.6.1)	Graphpad software	N/A
ClusterProfiler	N/A	https://github.com/YuLab-SMU/clusterProfilerRRID:SCR_016884
FastQC	N/A	https://github.com/s-andrews/FastQC
Trim Galore	N/A	https://github.com/FelixKrueger/TrimGalore
limma	Ritchie et al., 2015^55^	https://bioconductor.org/packages/release/bioc/html/limma.html
tximport	Soneson et al., 2015^56^	github.com/thelovelab/tximport
Salmon	N/A	https://github.com/COMBINE-lab/salmon RRID:SCR_017036
Scone	Cole et al., 2019^57^	https://github.com/YosefLab/scone
SCnorm	Bacher et al., 2017^58^	https://github.com/rhondabacher/SCnorm
SCRAN	Lun et al., 2016^59^	https://github.com/elswob/SCRAN RRID:SCR_016944
Seurat v4	Hao et al., 2021^60^	https://satijalab.org/seurat/

### Experimental model and study participant details

#### Animal studies

B6 (C57BL/6J, RRID:MGI:3028467) mice were purchased from Charles River Laboratories. F5 TCR-transgenic mice on a B6 background (B6/J-Tg(CD2-TcraF5,CD2-TcrbF5)1Kio/Jmar)^61^ were crossed with B6-Ptprc^em(K302E)Jmar^/J)^62^ to obtain F5 x CD45.1 mice (B6-^Ptprcem(K302E)Jmar^/J-Tg(CD2-TcraF5,CD2-TcrbF5)1Kio/Jmar). B6 x Ly5a were obtained by crossing B6 mice with Ly5a mice. Mice were bred and housed under specific pathogen-free conditions in the AniRA-PBES animal facility (Lyon, France). Both males and females were used. All experiments were approved by our local ethics committee (CECCAPP, Lyon, France), and accreditations were obtained from governmental agencies (APAFIS#20005–2019030410254161 and APAFIS #48159–2024010816529153).

### Method details

#### Immunizations

The recombinant vaccinia virus (VV) expressing the NP68 epitope (VV-NP68) was engineered from VV (strain Western Reserve) by Dr. Denise Yu-Lin Teoh in Pr. Sir Andrew McMichael’s laboratory at the MRC (Human Immunology Unit, Institute of Molecular Medicine, Oxford, UK). The recombinant influenza virus strain WSN encoding the NP68 epitope (Flu-NP68) was produced by reverse genetics from the A/WSN/33 H1N1 strain.^61^ EL4 lymphoma cell line expressing the NP68 epitope (EL4-NP68) was provided by Dr. T.N.M. Schumacher. Naive F5 x CD45.1 cells (2.10^5^ under 200 μL) were transferred by intravenous (i.v.) injection into B6 hosts. The next day, recipient mice were inoculated intranasally (i.n.) with VV-NP68 (2.10^5^ pfu under 20 μL) or subcutaneously (s.c.) with EL4-NP68 cells (2.,5.10^6^ under 200 μL). For recall experiments, more than 30 days post-challenge (dpc), mice received a second immunization with either VV-NP68 or EL4-NP68, similar to the primary challenge. For protection experiments mice were infected i.n. with a lethal dose of Flu-NP68 virus (2.10^6^ TCID50 under 20 μL). The fraction of surviving animals was measured daily for 10 days, mice that lost more than 20% of their initial body weight were euthanized. In experiments designed to evaluate the impact of tumor exposure duration on the phenotype of memory cells, naive F5 cells (B6 background, 2.10^5^ under 200 μL) were transferred by i.v. injection into B6 x Ly5a hosts. The next day, recipient mice were inoculated i.n. with VV-NP68 (2.10^5^ pfu under 20 μL) or s.c. with EL4-NP68 cells (2.,5.10^6^ under 200 μL). Three days post-immunization, naive F5 x CD45.1 cells (2.10^5^ under 200 μL) were transferred by i.v. injection.

#### Sample collection and flow cytometry analysis

Mice were bled at intervals of at least 7 days or euthanized for organ collection. In some experiments, for the detection of circulating cells, mice received an i.v. injection of an anti-CD8a-BUV395 antibody (1 μg under 200 μL, BD Biosciences, RRID: AB_2732919), 3 min prior euthanasia. Spleens and dLN were mechanically disrupted and filtered through a sterile 100 μm nylon mesh filter. Lungs were dissociated using the Lung Dissociation kit (Miltenyi Biotech, RRID:SCR_020286) and a gentleMACS Dissociator (Miltenyi Biotech, RRID:SCR_025922), according to manufacturer’s instructions. Blood volume was precisely measured and blood cells were enumerated using FlowCount fluorospheres (Beckman Coulter, 7547053).

Single cell suspensions were first incubated with efluor780-coupled Fixable Viability Dye (ThermoFischer Scientific or TFS, RRID: AB_469584) for 20 min at 4°C. Nonspecific binding was then blocked with Fc-blocking antibody (Ab) 2.4G2 (home-made supernatant) for 10 min at 4°C. Surface staining was performed with an appropriate mixture of Ab diluted in staining buffer (PBS [TFS, 14190169] supplemented with 1% fetal calf serum [Biowest, S1810-500] and 0.09% NaN3 [Sigma-Aldrich, S2002-100G] for 30 min at 4°C. For intracellular staining, cells were fixed and permeabilized, according to the manufacturer’s instructions, using either CytoFix/CytoPerm buffer (BD Biosciences, 554714) for cytokines staining or Fixation/Permeabilization buffer from the Foxp3 Transcription Factor Staining Buffer Kit (eBioscience, 00-5523-00) for transcription factors staining. Fixed cells were then stained with an appropriate mixture of intracellular Ab for 30 min at 4°C. All analyses were performed using a BD LSRFortessa cell analyzer (BD Biosciences) and further analyzed using FlowJoTM v10 software (BD Biosciences, RRID:SCR_008520). All antibodies are listed in the Table S3.

#### Cell culture

The EL4-NP68 cells line was maintained for a maximum of 2 weeks at 37°C in a 5% CO2 incubator and cultured in complete RPMI medium, consisting of RPMI 1640 medium with GlutaMAX Supplement (TFS, 61870044) supplemented with 10% Fetal Calf Serum (BioWest, S1810-500), 50 μg/mL gentamicin (TFS, 15750094), 10 mM HEPES buffer (TFS, 15630056) and 50 μM 2-Mercaptoethanol (50 μM, TFS, 31350010). Murine primary cells were cultured in DMEM medium consisting with 4.5 μg/mL of glucose and GlutaMAX supplement (TFS, 61965059), supplemented with 6% Fetal Calf Serum (BioWest, S1810-500), MEM Non-Essential Amino Acids Solution (TFS, 11140035), 50 μg/mL gentamicin (TFS, 15750094), 10 mM HEPES buffer (TFS, 15630056) and 50 μM 2-Mercaptoethanol (50 μM, TFS, 31350010), and maintained at 37°C in a 7% CO2 incubator.

#### *In vitro* CD8^+^ T cells peptide-stimulation

Splenocytes were stimulated with 10 nM NP68 peptide (ASNENMDAM, Proteogenix) for 1 to 24 h. GolgiStop (BD Biosciences, 554724) was added during the last 4 h of culture for intracellular cytokine detection. The measurement of inhibitory receptors (PD-1 and TIM3) and CD69 was performed in absence of GolgiStop, as it blocks the expression of these markers at the cell surface. For the dose-response experiments, splenocytes were stimulated with NP68 peptide at a dose ranging from 10^−3^ to 10 nM, for 4 h in the presence of GolgiStop.

#### *In vitro* proliferation assay

Peptide-loaded bone marrow-derived dendritic cells (BMDC) were used to activate CD8^+^ T cells. Bone marrow cells were isolated by flushing femurs of B6 mice with complete RPMI medium. Cells were filtered through a 100-μm nylon cell strainer and cultured at 2.10^6^ cells/mL in complete RPMI medium supplemented with 100 ng/mL recombinant human Flt3l (Amgen). After 7 days, NP68 peptide (20 nM) and CpG ODN 1826 (2 mg/mL, InvivoGen, tlrl-1826) were added to the BMDC and cultured overnight before being washed and used for CD8^+^ T cells activation. Total CD8^+^ T cells were enriched from splenocytes using an autoMACS Pro Separator (Miltenyi Biotec, RRID:SCR_018596) with the CD8a+ T cell isolation kit (Miltenyi Biotec, 130-104-075) according to the manufacturer’s instructions. Enriched CD8^+^ T cells were then labeled with CTV (CellTrace Violet, 2.5 mM, TFS, C34557) according to the manufacturer’s instructions. CTV-labelled CD8^+^ T cells and NP68-loaded BMDC were co-cultured in a 1:1 ratio in the presence of murine 5% rIL-2 supernatant (corresponding to a final concentration of 11.5 ng/mL) for 4 days. Cells were then stimulated with NP68 (10 nM) for 4 h in the presence of GolgiStop. The number of cells was determined using FlowCount fluorospheres (Beckman Coulter, 7547053). The expansion index was calculated as follows: Number of F5 CD8^+^ T cells at the end of the culture/number of F5 CD8^+^ T cells stimulated on day 0.

#### *In vivo* cytotoxicity assay

Total splenocytes were incubated in medium with or without 10 nM NP68 for 2h and labeled with respectively CTV (TFS, C34557) or CFSE (TFS, C34554). A 1:1 ratio of NP68-loaded splenocytes: control splenocytes (2.10^6^ cells) was injected i.v. into naive or challenged mice. After 6, 16 or 44h, spleens were collected and the ratio of NP68-loaded splenocytes/control splenocytes was evaluated by flow cytometry for naive or challenged mice. The percentage of *in vivo* killing in individual challenged mice was calculated using the mean ratio of NP68-loaded splenocytes/control splenocytes in naive mice, as follows:

$$100-\left[100{*\left[\frac{\%\;\text{CTV}\_\text{NP}68-\text{loaded}\;\text{splenocytes}}{\%\;{\text{CFSE}}_{-}\text{control}\;\text{splenocytes}\;}\right]}^{\begin{array}{c} \text{Challenged}\\ \text{mice} \end{array}}/{\text{mean}\left[\frac{\%\;\text{CTV}\_\text{NP}68-\text{loaded}\;\text{splenocytes}}{\%\;{\text{CFSE}}_{-}\text{control}\;\text{splenocytes}\;}\right]}^{\begin{array}{c} \text{Naive}\\ \text{mice} \end{array}}\right]$$

#### ELISA

The concentration of IFNγ in the supernatant was quantified using ELISA MAX Standard Set for mouse IFNγ (BioLegend, RRID:AB_2893366) following manufacturer’s instructions. Absorbance was measured at 450 nm using an Infinite 200 microplate reader (TECAN, RRID:SCR_020543). The IFNγ concentration was normalized to the number of F5 cells per well on day 0 and expressed in ng/mL per 10^5^ F5 cells.

#### qRT-PCR

The VV-NP68 viral load was determined in the lungs after DNA extraction. The lungs were directly frozen in nitrogen and stored at −80°C before DNA extraction. The lungs were homogenized in 150 μL of PBS using a Precellys 24 tissue homogenizer (Bertin Technologies, RRID:SCR_022979) 1 for 15 s at 4 m/s. Total DNA was extracted from cell lysates using NucleoSpin Tissue kit (Macherey Nagel, 740952.50).

qPCR was performed in duplicate for each gene using the StepOnePlus Real-Time PCR System (TFS, RRID:SCR_015805) and the Platinum SYBR Green qPCR SuperMix-UDG with ROX (TFS, 11744100). Individual data were normalized to HPRT mRNA, by calculating the ΔCt (median Ct (gene) - median Ct (HPRT)). Primers (Sigma–Aldrich) were used as follows:

VV-HA-Forward CATCATCTGGAATTGTCACTACTAAA; VV-HA-Reverse ACGGCCGACATATAATTAATGC; HPRT-Forward AAAGACTTGCTCGAGATG; HPRT-Reverse TAATGTAAT-CCAGCAGGTC.

#### Single-cell RNA sequencing

CD45.1 + F5 memory cells were sorted from the spleens of VV- or EL4-immunised mice 9 weeks after the primary challenge. Naive F5 cells were sorted from the spleen of F5 x CD45.1 mice. Enriched CD8^+^ T cells (see in vitro proliferation assay) were stimulated (Vir-CD8^+^ _R, Tum-CD8^+^ _R, naive_R) or not (Vir-CD8^+^, Tum-CD8^+^, Naive) with NP68 peptide (10 nM) for 2h at 37°C. F5 cells were then single-cell sorted according to their viability and their expression of CD45.1, CD8a, and CD44, into a 96-well plate containing 4 μL of lysis solution (PBS, 0.04% Triton 0.4%, 2 U/μL RNaseOut [TFS, 10777019], 2.5 μM of poly(T) reverse transcription primers [TFS, N8080127] and 2.5 mM dNTPs [TFS, R0192]), on a FACS Aria II (BD Biosciences).

Library construction was performed following the Smart-seq2 protocol.^63^ Briefly, mRNA was reverse transcribed into cDNA and pre-amplified by PCR. cDNAs was then fragmented by tagmentation and Illumina sequencing adaptors containing indexes allowing for cell identification were added. The cDNA was amplified by PCR. Each 96-cells libraries were pooled together and both quality and quantity were tested using a DNA high sensitivity D1000 ScreenTape (Agilent, 5067–5584) on Tapestation 4200 (Agilent, RRID:SCR_018435). Libraries were sequenced on an Illumina HiSeq in paired-ends (2 x 150 bp) at an average sequencing depth of ∼1M reads/cells.

#### scRNA-seq analysis

Quality control of the raw data was performed using FastQC. The Nextera Transposase sequence and low-quality bases were trimmed using Trim Galore. Transcript expression was quantified using Salmon (RRID:SCR_017036) and version M23 of GENCODE mouse genome and transcriptome (RRID:SCR_014966). The gene/count matrix was generated with tximport^56^ (RRID:SCR_016752). Cells were filtered using the *metric_sample_filter* function implemented in SCONE package.^57^ For each criterion (the number of reads, the number of detected genes and the areas under the false negative rate (FNR) curve employing housekeeping genes), values greater than three median absolute deviations from the median were excluded. Moreover, genes expressed in fewer than three cells were removed.

Then, the dataset consisting of 476 cells was normalized using SCnorm^58^ and variable features (HVG) were selected based on variance modeling statistics from the *modelGeneVar* function in Scran (RRID:SCR_016944).^59^ The top 10% of genes with positive biological components were used for further analysis. The log-normalized expression values of the 570 HVGs were used for advanced analysis.

The top eight principal components were selected for UMAP and clustering analysis. Clustering was performed by applying the Louvain algorithm included in the Seurat package V4.^60^ The top 20 differentially expressed genes (DEG) for each group of cells were determined using the *FindAllMarkers* function (logfc.threshold = 0.5 and min.diff.pct = 0.25) from the Seurat package (RRID:SCR_007322). Differential expression analysis was performed using Limma^55^ and applied to all genes and not just on HVGs. Heatmap, dot plot and violin plot were generated using the Seurat function *DoHeatmap*, *DotPlot* and *VlnPlot* respectively. Gene ontology (GO) enrichment analysis selecting biological process was performed using the *enrichGO* function implemented in the ClusterProfiler package (RRID:SCR_016884). Visualization was made with the enrichplot package.

### Quantification and statistical analysis

The sample size for each experiment was determined to be sufficiently large to guarantee the validity of the statistical results. Statistical analyses were performed using Graphpad software Prism 10 (RRID:SCR_000306). Groups comparison was performed using the Mann-Whitney *t* test, one-away ANOVA or two-way ANOVA, as indicated.
