# Supplementary material for: Transient tumor exposure induces persistent functional defects in memory CD8+ T cells
Source: iScience. 2026 Apr 1;29(5):115556. doi: 10.1016/j.isci.2026.115556 (PMC13098610; doi:10.1016/j.isci.2026.115556)
Supplement: Document S1. Figures S1–S5 and Tables S1–S3 [file mmc1.pdf]

iScience, Volume *29*

Supplemental information

Transient tumor exposure induces persistent functional defects in memory $CD8^+$ T cells

Daphné Laubreton, Margaux Prieux, Sophia Djebali, Maxence Dubois, Simon De Bernard, Olivier Gandrillon, Christophe Arpin, and Jacqueline Marvel

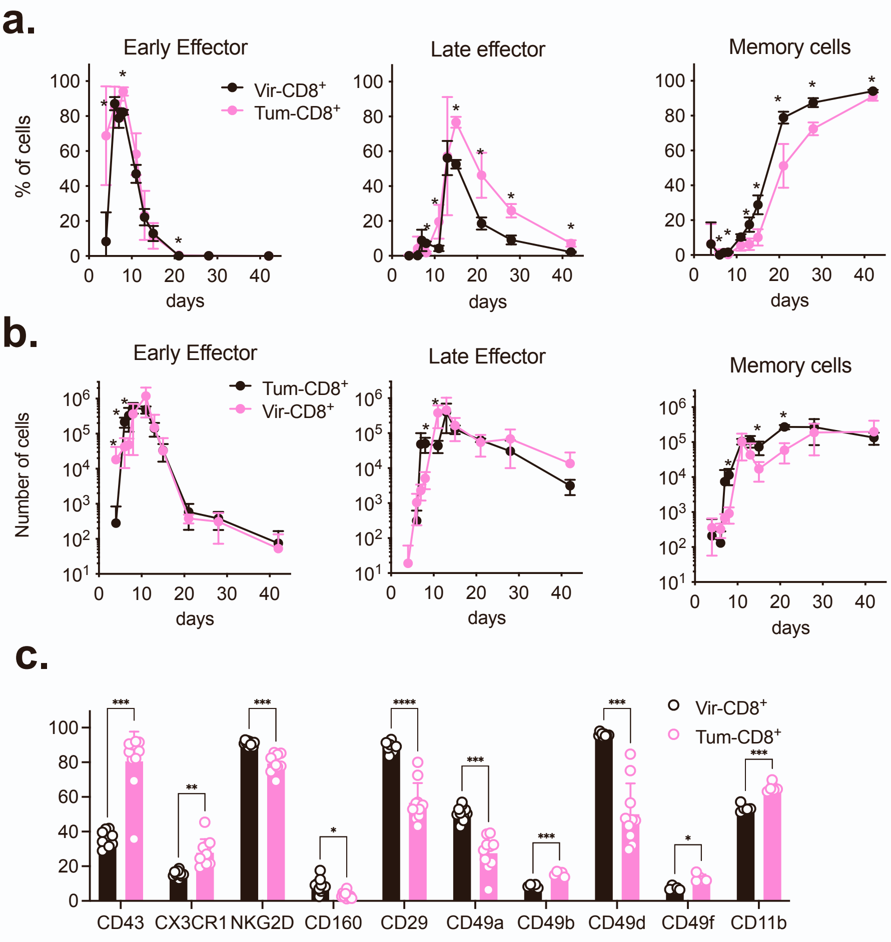

Supplementary Figure 1: Delayed memory cells generation following transient tumoral challenge

Naive F5 x CD45.1 cells (2.10⁵) were i.v. transferred in B6 mice 1-day prior immunisation with VV-NP68 (i.n., 2.10⁵ pfu) or EL4-NP68 cells (s.c., 2,5.10⁶ cells). (**a-b**) The percentages (**a**) and the numbers (**b**) of Early effectors, Late effectors or Memory cells within Vir-CD8⁺ and Tum-CD8⁺ cells were determined over time in the blood by flow cytometry, using Ki67 and Bcl2 labelling. (**c**) Markers with statistical significant differences identified in Fig.1e are represented. Statistical significance of differences was determined using a two-way ANOVA (* p <0.05, ** p <0.01, *** p <0.001, **** p <0.0001). Data are represented as mean ± SD and are representative of 3 independent experiments.

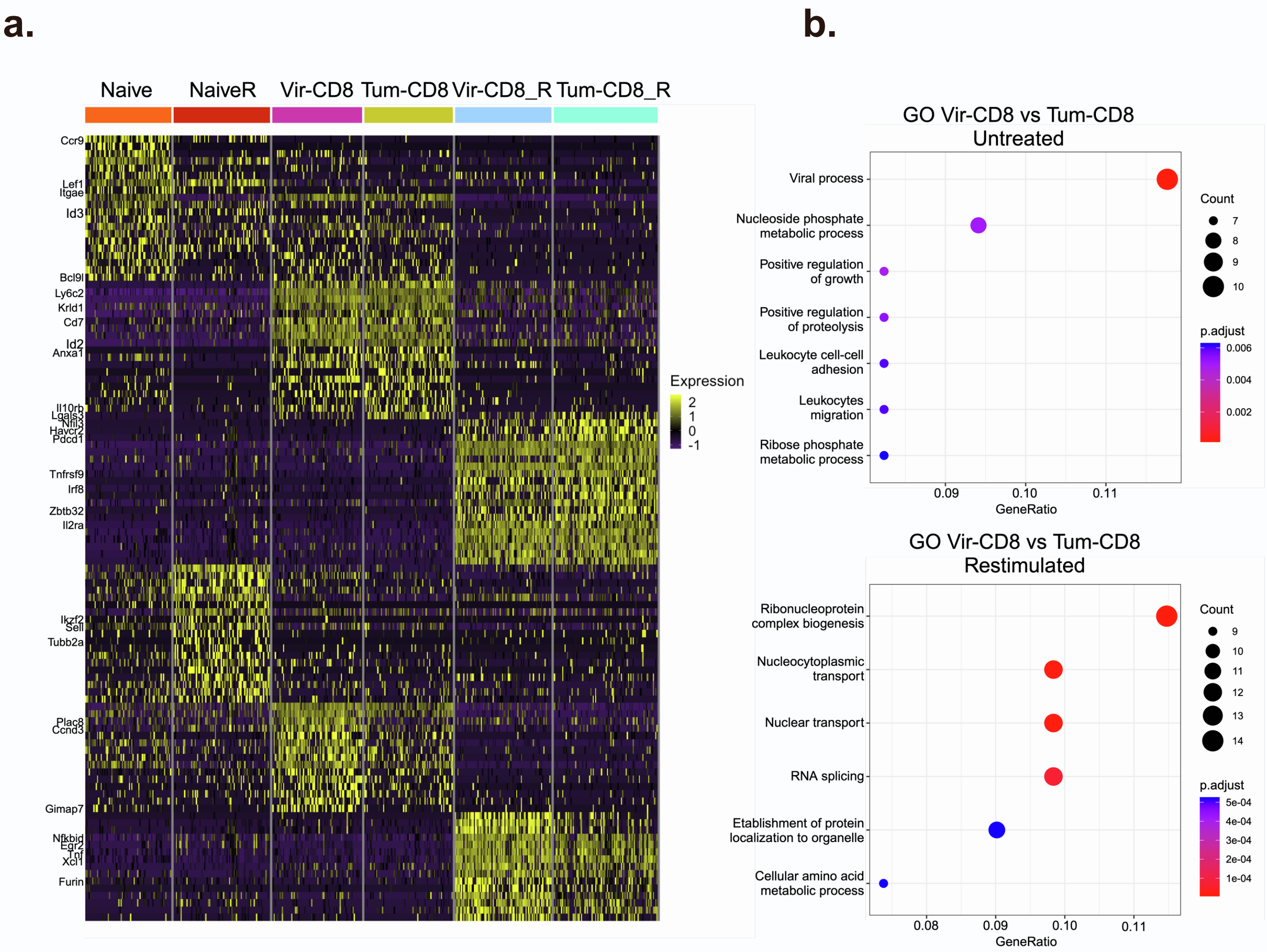

Supplementary Figure 2: Genes differentially expressed between Tum-CD8⁺ and Vir-CD8⁺ memory cells

Transcriptomic analysis of and Vir-CD8⁺ memory cells was performed as described in Figure 1. (**a**) Heatmap of the topTum-CD8⁺ 20 markers for each group. (**b**) Top GO analysis of biological processes of DEGs in Vir-CD8⁺ compared to Tum-CD8⁺ cells untreated or restimulated. Node size represents the number of genes, and color intensity corresponds to the adjusted p-value.

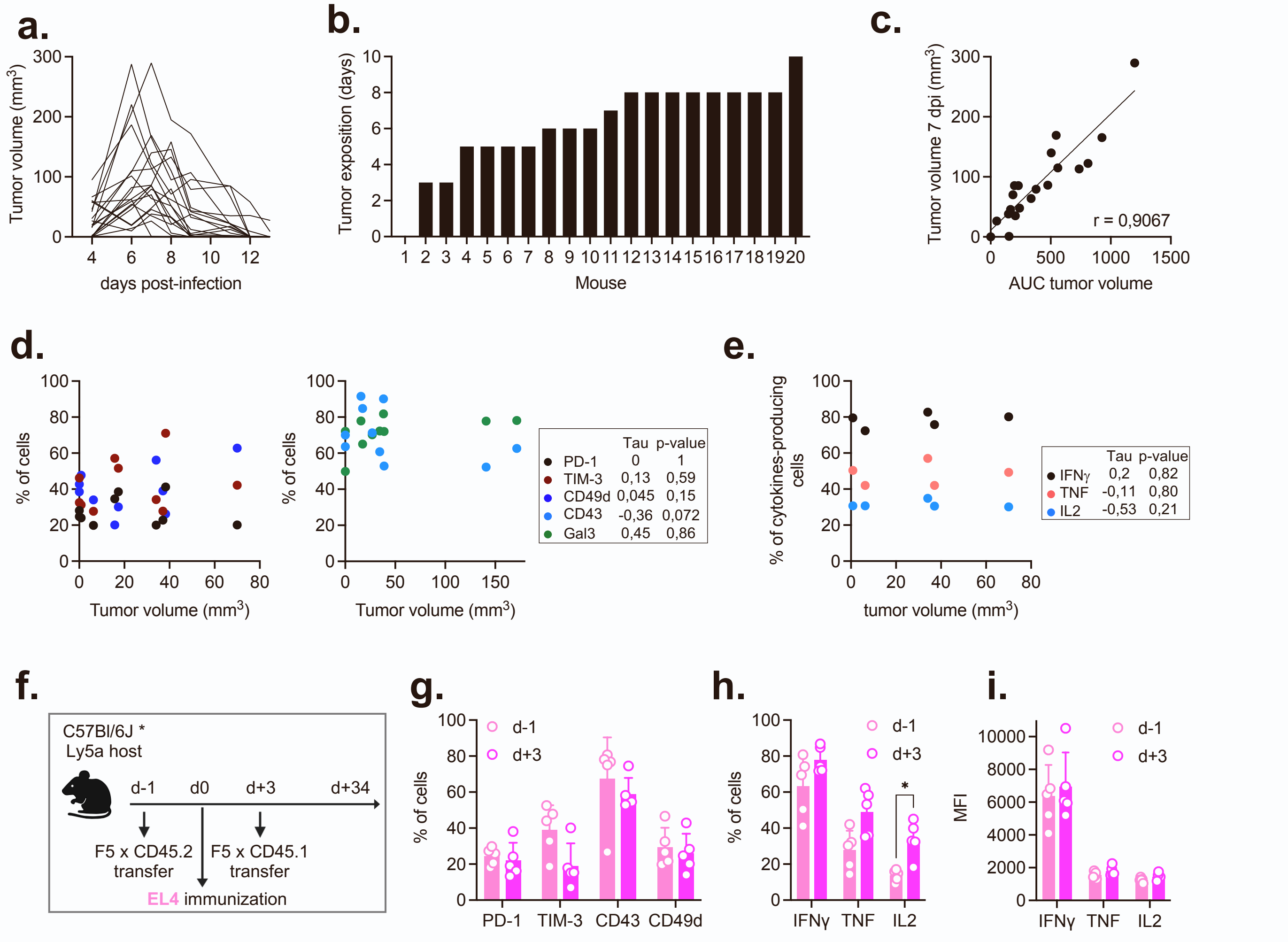

Supplementary Figure 3 : Impact of tumor growth and exposure duration on the phenotype and function of Tum-CD8⁺ cells

(**a-e**) Naive F5 x CD45.1 cells (2.10^5) were i.v. transferred in B6 mice 1-day prior immunisation with EL4-NP68 cells (s.c., 2,5.10^6 cells). (**a**) Tumor volume (mm^3) was assessed by measuring its length, width and thickness over time and represented for each individual mouse. (**b**) The duration of tumor exposition was calculated for each individual mouse. (**c**) The correlation between the AUC of the curve of tumor growth and the tumor volume at 7 dpi was determined. (**d-e**) The correlation between (**d**) the expression of PD-1, TIM-3, CD43 and CD49d or (**e**) the cytokine production in response to NP68 stimulation, with the tumor volume at 7 dpi were assessed using Kendall's non-parametric rank correlation test (Kendall's tau). Each point represents an individual mouse. Tau values and corresponding p-values are reported for each marker. (**f**) Naive F5 x CD45.2 cells (2.10^5) were i.v. transferred in B6 x Ly5a mice 1-day prior immunisation with EL4-NP68 cells (s.c., 2,5.10^6 cells). Then naive F5 x CD45.1 cells (2.10^5) were i.v. transferred at 3 dpi. (**g**) The expression of PD-1, TIM-3, CD43 and CD49d was measured at the surface of CD8 memory cells at 34 dpi by flow cytometry. (**h-i**) At 34 dpi, splenocytes were stimulated with NP68 peptide (10 nM) for 4 hours, and the expression of IFNγ, TNF and IL2 by memory CD8 T cells was measured by flow cytometry, and expressed in percentages (**h**) or MFI (**i**). The statistical significance of differences was determined with Mann-Whitney test (* $p < 0.05$). Data are represented as mean ± SD (n=5 mice per group).

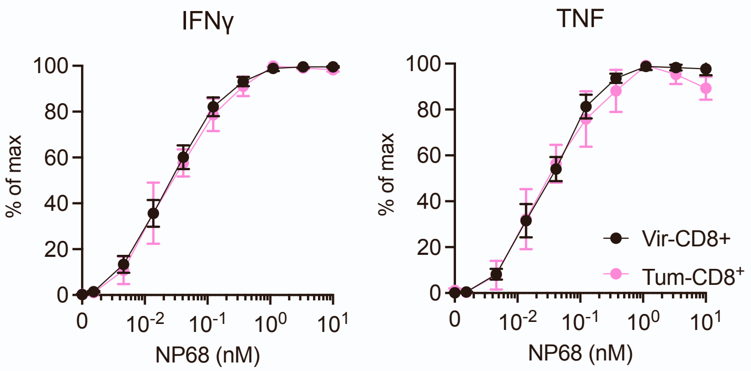

Supplementary figure 4 : IFNγ and TNF dose response to NP68 stimulation

Naive F5 x CD45.1 cells (2.10^5) were i.v. transferred in B6 mice 1-day prior immunisation with VV-NP68 (i.n., 2.10^5 pfu) or EL4-NP68 cells (s.c., $2{,}5.10^6$ cells). At 30 dpi, F5 memory cells were restimulated with various doses of NP68 for 4h in the presence of GolgiStop. The production of IFNγ and TNF was measured by flow cytometry and expressed in percentage of maximal production. Data are represented as mean ± SD (n= 5 mice per group) and are representative of 3 independents experiments.

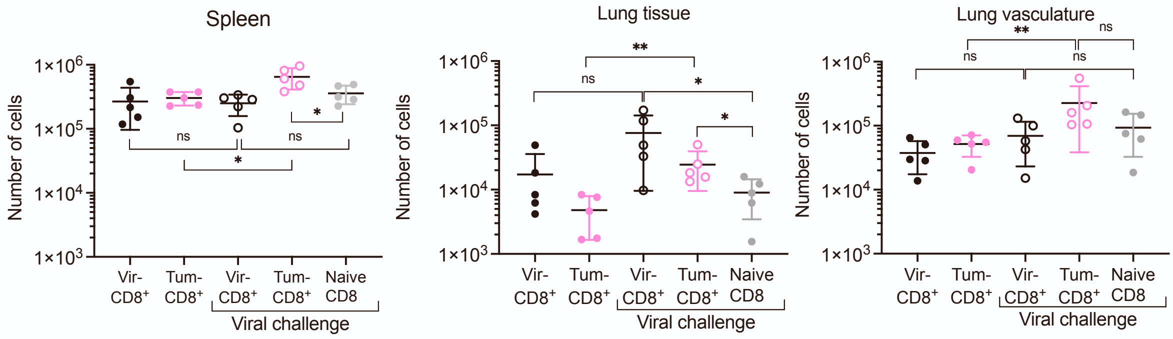

Supp Figure 5 : A transient tumoral challenge is sufficient to alter the protection capacity of F5 memory cells

Naive F5 x CD45.1 cells (2.10^5) were i.v. transferred in B6 mice 1-day prior immunisation with VV-NP68 (i.n., 2.10^5 pfu) or EL4-NP68 cells (s.c., $2,5.10^6$ cells). At 30 dpi, Vir- or Tum-challenged mice were infected with VV-NP68. Six days post-challenge, mice received an i.v. injection of anti-CD8 antibody to label circulating cells. The number of CD8 memory cells in the spleen, lung tissue and lung vasculature were determined. The statistical significance of differences was determined using a one-way-ANOVA (* $p< 0.05$, ** $p< 0.01$). Data are represented as mean ± SD (n=5 mice per group).

Supplementary Table 1 : Differentially expressed genes between Vir-CD8 and Tum-CD8

Gene	Comparison	logFC	AveExpr	P.Value	adj.P.Val
Lgals3	V-CD8 vs T-CD8 (quiescent)	1,625254	1,915122	7,74E-06	0,003988
Capg	V-CD8 vs T-CD8 (quiescent)	1,472966	1,720957	9,96E-06	0,004906
X4930453N24Rik	V-CD8 vs T-CD8 (quiescent)	-1,014758	1,229679	0,000406	0,038981
Creld2	V-CD8 vs T-CD8 (quiescent)	-1,021205	0,814707	0,000122	0,02092
Phb	V-CD8 vs T-CD8 (quiescent)	-1,02711	1,721956	0,000485	0,043425
Farsb	V-CD8 vs T-CD8 (quiescent)	-1,033709	1,219465	0,000496	0,043794
Ctsd	V-CD8 vs T-CD8 (quiescent)	-1,039768	4,727457	0,000171	0,025011
Shmt2	V-CD8 vs T-CD8 (quiescent)	-1,040498	1,240223	0,000314	0,033775
Tspan3	V-CD8 vs T-CD8 (quiescent)	-1,040634	0,666225	5,46E-05	0,012418
Acly	V-CD8 vs T-CD8 (quiescent)	-1,0426	1,409394	0,000537	0,045667
Naa10	V-CD8 vs T-CD8 (quiescent)	-1,043097	0,976588	2,48E-05	0,009164
Snx3	V-CD8 vs T-CD8 (quiescent)	-1,043746	1,80943	0,000611	0,048167
Slc25a11	V-CD8 vs T-CD8 (quiescent)	-1,045859	1,346305	0,000583	0,046907
Tmem50a	V-CD8 vs T-CD8 (quiescent)	-1,051907	2,977549	0,000237	0,031128
Zfp263	V-CD8 vs T-CD8 (quiescent)	-1,058239	1,064446	0,000269	0,031331
Ppib	V-CD8 vs T-CD8 (quiescent)	-1,060374	2,368162	0,000688	0,049707
Il21r	V-CD8 vs T-CD8 (quiescent)	-1,064968	1,403454	0,000668	0,049453
Ist1	V-CD8 vs T-CD8 (quiescent)	-1,065415	1,20273	0,000264	0,031331
Cdc42	V-CD8 vs T-CD8 (quiescent)	-1,07222	4,856084	2,42E-05	0,009164
Fam173a	V-CD8 vs T-CD8 (quiescent)	-1,073231	1,655382	0,000135	0,022208
Serinc3	V-CD8 vs T-CD8 (quiescent)	-1,073794	2,424404	0,000266	0,031331
Sptssa	V-CD8 vs T-CD8 (quiescent)	-1,075221	1,912501	0,000298	0,032376
Mbnl1	V-CD8 vs T-CD8 (quiescent)	-1,075841	4,285004	1,34E-09	7,92E-06
Sp100	V-CD8 vs T-CD8 (quiescent)	-1,07599	3,525112	0,000353	0,035719
Rae1	V-CD8 vs T-CD8 (quiescent)	-1,078329	1,043214	0,000249	0,031331
Twf2	V-CD8 vs T-CD8 (quiescent)	-1,095694	1,456291	0,000298	0,032376
Atp6ap1	V-CD8 vs T-CD8 (quiescent)	-1,100259	1,53017	0,000616	0,048167
Atp5c1	V-CD8 vs T-CD8 (quiescent)	-1,11384	2,933699	0,000339	0,035118
Myc	V-CD8 vs T-CD8 (quiescent)	-1,114704	1,77144	0,000291	0,032376
Pim2	V-CD8 vs T-CD8 (quiescent)	-1,125613	0,99561	3,2E-05	0,010641
Ech1	V-CD8 vs T-CD8 (quiescent)	-1,126702	1,775793	0,000556	0,046626
Pcmt1	V-CD8 vs T-CD8 (quiescent)	-1,130677	1,166088	6,5E-05	0,014242
Nat8f4	V-CD8 vs T-CD8 (quiescent)	-1,14124	2,939773	0,000544	0,045928
Tmem59	V-CD8 vs T-CD8 (quiescent)	-1,141573	2,701004	0,000221	0,02973
Gimap7	V-CD8 vs T-CD8 (quiescent)	-1,145071	1,174652	5,33E-05	0,012368
Cdc42se2	V-CD8 vs T-CD8 (quiescent)	-1,14815	1,686899	0,00067	0,049453
Dynll1	V-CD8 vs T-CD8 (quiescent)	-1,149407	2,433257	0,000163	0,024672
Fli1	V-CD8 vs T-CD8 (quiescent)	-1,150509	1,463281	0,000184	0,025898
Serpina3g	V-CD8 vs T-CD8 (quiescent)	-1,152119	0,781852	3,76E-05	0,011583
Isca1	V-CD8 vs T-CD8 (quiescent)	-1,16906	1,014166	2,94E-05	0,010226
Itgb2	V-CD8 vs T-CD8 (quiescent)	-1,169518	3,729529	0,000277	0,031494
Mat2b	V-CD8 vs T-CD8 (quiescent)	-1,169826	1,827108	0,000345	0,035203
Ucp2	V-CD8 vs T-CD8 (quiescent)	-1,171368	4,058831	4,57E-05	0,011583
Bsg	V-CD8 vs T-CD8 (quiescent)	-1,174168	2,1557	0,000244	0,031331
Epsti1	V-CD8 vs T-CD8 (quiescent)	-1,183624	3,52151	2,16E-05	0,0085
Eno1	V-CD8 vs T-CD8 (quiescent)	-1,185594	4,482357	0,000197	0,027149
Serp1	V-CD8 vs T-CD8 (quiescent)	-1,187483	1,833831	0,000264	0,031331
Gramd1a	V-CD8 vs T-CD8 (quiescent)	-1,197469	2,196526	0,000482	0,043425
Fam49b	V-CD8 vs T-CD8 (quiescent)	-1,199567	2,408315	0,000363	0,036089
Dok2	V-CD8 vs T-CD8 (quiescent)	-1,206592	1,626643	0,000253	0,031331
Cops6	V-CD8 vs T-CD8 (quiescent)	-1,206967	1,776074	0,000256	0,031331
Rbm42	V-CD8 vs T-CD8 (quiescent)	-1,207614	1,649025	0,000273	0,031331
Dpp4	V-CD8 vs T-CD8 (quiescent)	-1,217702	1,987492	0,000161	0,024661
Gm12248	V-CD8 vs T-CD8 (quiescent)	-1,222529	1,372593	4,61E-05	0,011583
Klf12	V-CD8 vs T-CD8 (quiescent)	-1,223683	1,242841	4,2E-05	0,011583
Eif4a1	V-CD8 vs T-CD8 (quiescent)	-1,227522	3,685026	0,000147	0,023565
Zfp207	V-CD8 vs T-CD8 (quiescent)	-1,228422	2,112972	9,28E-05	0,017825
Tbc1d10c	V-CD8 vs T-CD8 (quiescent)	-1,229169	4,407461	0,000158	0,024661
Akr1a1	V-CD8 vs T-CD8 (quiescent)	-1,247927	3,018386	0,000623	0,048167
Atp5f1	V-CD8 vs T-CD8 (quiescent)	-1,254725	3,064336	0,000318	0,033851
Olfr461	V-CD8 vs T-CD8 (quiescent)	-1,268711	2,478085	0,000244	0,031331
Traf3ip3	V-CD8 vs T-CD8 (quiescent)	-1,277229	2,091609	4E-05	0,011583
Tmed9	V-CD8 vs T-CD8 (quiescent)	-1,281349	1,856452	1,82E-05	0,007425
Rnf167	V-CD8 vs T-CD8 (quiescent)	-1,282201	1,802119	4,84E-05	0,011583
Sirt7	V-CD8 vs T-CD8 (quiescent)	-1,299067	2,016665	0,00013	0,021608
Pgk1	V-CD8 vs T-CD8 (quiescent)	-1,307684	2,010788	0,000401	0,038887
B4galnt1	V-CD8 vs T-CD8 (quiescent)	-1,312988	3,590971	0,000165	0,024672
Prelid1	V-CD8 vs T-CD8 (quiescent)	-1,31579	2,911035	9,35E-05	0,017825
Ahsa1	V-CD8 vs T-CD8 (quiescent)	-1,325934	1,705994	4,82E-05	0,011583
Tagln2	V-CD8 vs T-CD8 (quiescent)	-1,341919	4,254439	7,76E-06	0,003988
Fubp1	V-CD8 vs T-CD8 (quiescent)	-1,345618	2,412103	0,000141	0,022779
Arhgef3	V-CD8 vs T-CD8 (quiescent)	-1,362457	1,885103	0,000159	0,024661
Mcm6	V-CD8 vs T-CD8 (quiescent)	-1,373709	1,255152	6,43E-06	0,00367
Cap1	V-CD8 vs T-CD8 (quiescent)	-1,395695	2,048531	2,19E-06	0,00158
Phyhd1	V-CD8 vs T-CD8 (quiescent)	-1,406496	1,616028	3,24E-05	0,010641
Dgka	V-CD8 vs T-CD8 (quiescent)	-1,416096	3,163159	4,84E-05	0,011583

Itga4	V-CD8 vs T-CD8 (quiescent)	-1,434479	2,630609	4,07E-05	0,011583
Dbnl	V-CD8 vs T-CD8 (quiescent)	-1,445548	1,795407	1,4E-05	0,006125
Prkar1a	V-CD8 vs T-CD8 (quiescent)	-1,489754	3,202676	4,59E-06	0,003016
Mob3a	V-CD8 vs T-CD8 (quiescent)	-1,504381	2,26307	1,72E-05	0,007267
Cnp	V-CD8 vs T-CD8 (quiescent)	-1,509945	2,816134	3,69E-05	0,011583
Ptbp1	V-CD8 vs T-CD8 (quiescent)	-1,511374	2,275024	1,14E-05	0,005373
Esyt1	V-CD8 vs T-CD8 (quiescent)	-1,544452	2,991743	6,52E-06	0,00367
Arhgap9	V-CD8 vs T-CD8 (quiescent)	-1,545016	2,596265	6,32E-06	0,00367
Msn	V-CD8 vs T-CD8 (quiescent)	-1,58116	3,787401	1,61E-06	0,001265
Ubc	V-CD8 vs T-CD8 (quiescent)	-1,619149	4,316413	1,24E-05	0,005622
Skap1	V-CD8 vs T-CD8 (quiescent)	-1,631548	2,912151	2,46E-07	0,000416
Stip1	V-CD8 vs T-CD8 (quiescent)	-1,63364	1,53207	5,55E-07	0,000621
Ltb	V-CD8 vs T-CD8 (quiescent)	-1,634619	3,872896	6,3E-07	0,000621
Vcp	V-CD8 vs T-CD8 (quiescent)	-1,806603	2,591343	4,76E-07	0,000621
Ccnd3	V-CD8 vs T-CD8 (quiescent)	-1,837476	3,282465	1,43E-06	0,001208
Sash3	V-CD8 vs T-CD8 (quiescent)	-1,846781	3,219753	6,83E-08	0,000162
Stat1	V-CD8 vs T-CD8 (quiescent)	-1,976783	2,375643	3,03E-08	8,94E-05
Plac8	V-CD8 vs T-CD8 (quiescent)	-2,313927	2,2654	8,6E-15	1,02E-10
Ubash3b	V-CD8 vs T-CD8 (restimulated)	1,701878	3,070981	8,06E-07	0,000288
Capg	V-CD8 vs T-CD8 (restimulated)	1,492867	2,061938	9,06E-06	0,001977
Icos	V-CD8 vs T-CD8 (restimulated)	1,417736	2,708953	0,000107	0,010817
Pdcd1	V-CD8 vs T-CD8 (restimulated)	1,398956	3,215749	0,000327	0,022459
Lgals3	V-CD8 vs T-CD8 (restimulated)	1,378564	1,746515	8E-05	0,009027
Havcr2	V-CD8 vs T-CD8 (restimulated)	1,355103	2,4084	7,52E-05	0,008727
Prf1	V-CD8 vs T-CD8 (restimulated)	1,34522	2,192506	0,000171	0,014477
Nfil3	V-CD8 vs T-CD8 (restimulated)	1,344468	2,565103	0,00015	0,01337
Ccr7	V-CD8 vs T-CD8 (restimulated)	1,299263	1,505029	0,000102	0,010335
Klrk1	V-CD8 vs T-CD8 (restimulated)	1,277448	3,118256	0,000828	0,042262
Spry1	V-CD8 vs T-CD8 (restimulated)	1,260515	2,381232	0,000374	0,024645
Cd9	V-CD8 vs T-CD8 (restimulated)	1,167128	2,472069	0,0002	0,015853
Bzw1	V-CD8 vs T-CD8 (restimulated)	-1,003264	4,262455	0,000153	0,013466
Paics	V-CD8 vs T-CD8 (restimulated)	-1,006487	1,485351	0,000531	0,03099
Cmtm6	V-CD8 vs T-CD8 (restimulated)	-1,008008	0,881435	9,36E-05	0,009895
Acly	V-CD8 vs T-CD8 (restimulated)	-1,009554	1,069382	0,000196	0,015579
Jak2	V-CD8 vs T-CD8 (restimulated)	-1,010391	1,634319	0,000963	0,047304
Tm9sf3	V-CD8 vs T-CD8 (restimulated)	-1,016455	1,080851	0,000177	0,014513
Slc1a5	V-CD8 vs T-CD8 (restimulated)	-1,017048	5,730134	1,19E-06	0,00039
Ccnl1	V-CD8 vs T-CD8 (restimulated)	-1,017908	3,03558	0,000725	0,038493
Ppp1r16b	V-CD8 vs T-CD8 (restimulated)	-1,022803	3,096219	2,39E-05	0,003884
Tex10	V-CD8 vs T-CD8 (restimulated)	-1,023928	1,2868	0,000204	0,016067
Celf1	V-CD8 vs T-CD8 (restimulated)	-1,024943	1,928587	0,000328	0,022459
Wsb1	V-CD8 vs T-CD8 (restimulated)	-1,028625	2,321395	0,000972	0,047543
Qrich1	V-CD8 vs T-CD8 (restimulated)	-1,028792	1,671103	0,000398	0,025682
Omt2a	V-CD8 vs T-CD8 (restimulated)	-1,032195	1,387011	0,000115	0,01145
Tgif2	V-CD8 vs T-CD8 (restimulated)	-1,034688	1,159843	0,00012	0,011909
Rab3gap1	V-CD8 vs T-CD8 (restimulated)	-1,035463	1,902575	1,71E-05	0,003068
Myo1g	V-CD8 vs T-CD8 (restimulated)	-1,036675	1,018385	0,000137	0,01245
Uspl1	V-CD8 vs T-CD8 (restimulated)	-1,039332	0,96245	3,34E-05	0,004743
Pprc1	V-CD8 vs T-CD8 (restimulated)	-1,04048	1,929712	0,000165	0,014291
Mak16	V-CD8 vs T-CD8 (restimulated)	-1,040722	2,882269	8,57E-05	0,009288
Vav1	V-CD8 vs T-CD8 (restimulated)	-1,041416	1,180492	8,63E-05	0,009288
Mafk	V-CD8 vs T-CD8 (restimulated)	-1,052306	1,729358	0,000634	0,035101
Rhbdf2	V-CD8 vs T-CD8 (restimulated)	-1,052335	1,433507	0,000536	0,031029
Arhgef2	V-CD8 vs T-CD8 (restimulated)	-1,053585	1,214316	4,68E-05	0,006175
Ppp2r1b	V-CD8 vs T-CD8 (restimulated)	-1,06553	1,367402	0,000172	0,014477
Surf6	V-CD8 vs T-CD8 (restimulated)	-1,066957	1,382366	8,04E-05	0,009027
Lars	V-CD8 vs T-CD8 (restimulated)	-1,070661	2,217871	0,000176	0,014513
Anapc16	V-CD8 vs T-CD8 (restimulated)	-1,070818	1,271655	3,64E-05	0,005053
Nabp1	V-CD8 vs T-CD8 (restimulated)	-1,076056	2,558261	0,000317	0,022351
Rnf4	V-CD8 vs T-CD8 (restimulated)	-1,076235	2,605037	0,00067	0,036748
Noc4l	V-CD8 vs T-CD8 (restimulated)	-1,083076	2,36562	0,000788	0,040563
Ftsj3	V-CD8 vs T-CD8 (restimulated)	-1,086725	3,159447	0,000266	0,019854
Ppp5c	V-CD8 vs T-CD8 (restimulated)	-1,088959	1,300589	3,22E-05	0,00469
Sun2	V-CD8 vs T-CD8 (restimulated)	-1,089968	1,341095	0,000166	0,014291
Tbk1	V-CD8 vs T-CD8 (restimulated)	-1,089996	0,902996	8,05E-07	0,000288
Hmgcr	V-CD8 vs T-CD8 (restimulated)	-1,093332	1,175875	7,05E-05	0,008451
Snrnp70	V-CD8 vs T-CD8 (restimulated)	-1,094063	3,172867	0,000443	0,027355
Thoc2	V-CD8 vs T-CD8 (restimulated)	-1,095036	2,126327	9,4E-05	0,009895
Cltc	V-CD8 vs T-CD8 (restimulated)	-1,096708	1,608014	0,000456	0,027693
Rbm38	V-CD8 vs T-CD8 (restimulated)	-1,100408	1,502794	3,87E-05	0,00531
Umps	V-CD8 vs T-CD8 (restimulated)	-1,103806	1,743802	0,000632	0,035101
Xpot	V-CD8 vs T-CD8 (restimulated)	-1,105575	1,64547	0,000269	0,019897
Vbp1	V-CD8 vs T-CD8 (restimulated)	-1,10631	1,608362	2,42E-05	0,003884
Birc3	V-CD8 vs T-CD8 (restimulated)	-1,106475	1,34223	6,37E-05	0,007906
Arcn1	V-CD8 vs T-CD8 (restimulated)	-1,106787	1,80825	0,00017	0,014477
Eif2s3x	V-CD8 vs T-CD8 (restimulated)	-1,108975	1,993892	0,0003	0,02169
Pknox1	V-CD8 vs T-CD8 (restimulated)	-1,109556	1,413502	0,000463	0,027985
Cand1	V-CD8 vs T-CD8 (restimulated)	-1,114534	1,051065	7,1E-05	0,008451
Klf10	V-CD8 vs T-CD8 (restimulated)	-1,114831	0,673116	1,42E-06	0,000453
Cdk6	V-CD8 vs T-CD8 (restimulated)	-1,116848	1,305119	0,000415	0,026264

Pwp2	V-CD8 vs T-CD8 (restimulated)	-1,11869	1,741376	0,000324	0,022459
Tsr1	V-CD8 vs T-CD8 (restimulated)	-1,120009	2,683147	0,000917	0,04554
Ddx5	V-CD8 vs T-CD8 (restimulated)	-1,125441	5,743774	9,97E-17	1,18E-12
X2410002F23Rik	V-CD8 vs T-CD8 (restimulated)	-1,128077	2,740836	0,000132	0,012319
Thoc1	V-CD8 vs T-CD8 (restimulated)	-1,133077	1,644783	2,44E-05	0,003884
Dhx15	V-CD8 vs T-CD8 (restimulated)	-1,134024	1,999719	0,000125	0,012156
Fmnl1	V-CD8 vs T-CD8 (restimulated)	-1,141177	1,707875	1,67E-05	0,003068
Ssr3	V-CD8 vs T-CD8 (restimulated)	-1,141758	1,585212	7,59E-05	0,008727
Cse1l	V-CD8 vs T-CD8 (restimulated)	-1,14946	1,160223	1,56E-05	0,002971
Ywhaq	V-CD8 vs T-CD8 (restimulated)	-1,151541	2,491422	0,000303	0,021763
Clic4	V-CD8 vs T-CD8 (restimulated)	-1,1583	1,688642	6,46E-05	0,007919
Aldh18a1	V-CD8 vs T-CD8 (restimulated)	-1,159389	1,947648	0,000175	0,014497
Sf3b3	V-CD8 vs T-CD8 (restimulated)	-1,175559	2,141295	0,000184	0,014889
Arfrp1	V-CD8 vs T-CD8 (restimulated)	-1,179335	2,277969	0,000315	0,022351
Plk3	V-CD8 vs T-CD8 (restimulated)	-1,180398	2,914916	0,000439	0,02722
Peli1	V-CD8 vs T-CD8 (restimulated)	-1,18485	3,09753	0,00025	0,018897
Myc	V-CD8 vs T-CD8 (restimulated)	-1,189493	4,580542	0,000124	0,012156
Wdr77	V-CD8 vs T-CD8 (restimulated)	-1,191779	3,131111	0,000597	0,033659
Brd2	V-CD8 vs T-CD8 (restimulated)	-1,196857	2,958358	7,32E-06	0,001661
Tagap	V-CD8 vs T-CD8 (restimulated)	-1,202955	4,74577	3,99E-05	0,005407
Bcat1	V-CD8 vs T-CD8 (restimulated)	-1,209736	1,885067	8,66E-05	0,009288
Tnpo3	V-CD8 vs T-CD8 (restimulated)	-1,211619	1,615353	4,7E-05	0,006175
Efr3a	V-CD8 vs T-CD8 (restimulated)	-1,215264	1,668529	0,000173	0,014477
Dop1b	V-CD8 vs T-CD8 (restimulated)	-1,220792	3,686133	0,000338	0,022898
Adss	V-CD8 vs T-CD8 (restimulated)	-1,234536	2,065609	4,77E-05	0,006175
Azin1	V-CD8 vs T-CD8 (restimulated)	-1,241437	1,72106	2,72E-05	0,004223
Plek	V-CD8 vs T-CD8 (restimulated)	-1,242621	3,533224	7,28E-05	0,008579
Dytn	V-CD8 vs T-CD8 (restimulated)	-1,243228	2,856108	1,72E-05	0,003068
Trav6d.5	V-CD8 vs T-CD8 (restimulated)	-1,248229	1,71133	3,01E-07	0,000122
Ddb1	V-CD8 vs T-CD8 (restimulated)	-1,2713	1,294369	1,1E-06	0,000378
Rgs3	V-CD8 vs T-CD8 (restimulated)	-1,277528	2,484921	3,39E-05	0,004764
Lrrfip1	V-CD8 vs T-CD8 (restimulated)	-1,280745	2,606961	0,000134	0,012319
Nup98	V-CD8 vs T-CD8 (restimulated)	-1,282827	1,862059	3,19E-06	0,000876
Fcrl6	V-CD8 vs T-CD8 (restimulated)	-1,284478	2,603864	1,82E-05	0,003199
Matr3	V-CD8 vs T-CD8 (restimulated)	-1,295247	2,646408	6,92E-06	0,001599
Smg5	V-CD8 vs T-CD8 (restimulated)	-1,295743	1,438339	1,28E-05	0,002506
Dkc1	V-CD8 vs T-CD8 (restimulated)	-1,297936	3,335999	0,000126	0,012167
Exoc5	V-CD8 vs T-CD8 (restimulated)	-1,299826	1,11894	1,91E-06	0,000564
Pum2	V-CD8 vs T-CD8 (restimulated)	-1,306247	2,010882	2,99E-05	0,004515
Ranbp2	V-CD8 vs T-CD8 (restimulated)	-1,318006	1,675236	3,77E-06	0,000966
Usp4	V-CD8 vs T-CD8 (restimulated)	-1,326002	1,388075	4,87E-06	0,001196
Map3k8	V-CD8 vs T-CD8 (restimulated)	-1,331345	0,803871	2,32E-08	1,95E-05
Stt3a	V-CD8 vs T-CD8 (restimulated)	-1,331378	2,720927	7,54E-06	0,001679
Tgoln1	V-CD8 vs T-CD8 (restimulated)	-1,331766	3,027251	2,66E-05	0,004184
Serinc3	V-CD8 vs T-CD8 (restimulated)	-1,332506	1,969945	1,12E-06	0,000378
Eif4g2	V-CD8 vs T-CD8 (restimulated)	-1,341741	3,744852	2,97E-08	2,19E-05
Ppat	V-CD8 vs T-CD8 (restimulated)	-1,360477	1,506466	9,98E-06	0,0021
Fgfr1op2	V-CD8 vs T-CD8 (restimulated)	-1,364336	1,824516	6,75E-06	0,001592
Canx	V-CD8 vs T-CD8 (restimulated)	-1,364969	2,644701	3,89E-06	0,000977
Eif4b	V-CD8 vs T-CD8 (restimulated)	-1,367981	2,768937	2,1E-05	0,00353
Gnai3	V-CD8 vs T-CD8 (restimulated)	-1,415543	1,796129	6,53E-06	0,001571
Mybbp1a	V-CD8 vs T-CD8 (restimulated)	-1,44432	3,184278	1,84E-07	8,66E-05
Ncf4	V-CD8 vs T-CD8 (restimulated)	-1,448289	2,621638	2,29E-06	0,00066
Nfkbid	V-CD8 vs T-CD8 (restimulated)	-1,448921	5,291483	1,38E-05	0,002662
Shmt1	V-CD8 vs T-CD8 (restimulated)	-1,45146	2,08079	9,75E-06	0,002091
Iars	V-CD8 vs T-CD8 (restimulated)	-1,477589	2,601367	2,8E-06	0,000787
Abce1	V-CD8 vs T-CD8 (restimulated)	-1,489158	2,390189	3,49E-06	0,000935
Hspa9	V-CD8 vs T-CD8 (restimulated)	-1,519815	4,338461	8,1E-09	7,96E-06
Luc7l	V-CD8 vs T-CD8 (restimulated)	-1,531679	2,07286	4,3E-07	0,000169
Cd274	V-CD8 vs T-CD8 (restimulated)	-1,596217	2,86478	3,58E-06	0,000938
Gm11634	V-CD8 vs T-CD8 (restimulated)	-1,642375	2,664737	1,68E-06	0,000509
Prkch	V-CD8 vs T-CD8 (restimulated)	-1,642722	2,071209	4,71E-08	3,27E-05
Caprin1	V-CD8 vs T-CD8 (restimulated)	-1,647674	2,822405	1,69E-07	8,32E-05
Rassf5	V-CD8 vs T-CD8 (restimulated)	-1,648602	2,788391	2,36E-07	0,000107
Ncoa4	V-CD8 vs T-CD8 (restimulated)	-1,655345	1,524243	5,6E-08	3,3E-05
Ehd1	V-CD8 vs T-CD8 (restimulated)	-1,674087	3,355148	6,66E-07	0,000253
Arhgef3	V-CD8 vs T-CD8 (restimulated)	-1,71322	2,994292	1,49E-06	0,000461
F2r	V-CD8 vs T-CD8 (restimulated)	-1,715879	1,852468	5,12E-08	3,3E-05
Ipo5	V-CD8 vs T-CD8 (restimulated)	-1,727781	2,786451	6,78E-08	3,8E-05
Trav12.1	V-CD8 vs T-CD8 (restimulated)	-1,738188	2,157945	4,46E-11	1,05E-07
Zbtb1	V-CD8 vs T-CD8 (restimulated)	-1,739948	1,829038	5,55E-08	3,3E-05
Cflar	V-CD8 vs T-CD8 (restimulated)	-1,741056	3,29425	1,58E-07	8,1E-05
Aars	V-CD8 vs T-CD8 (restimulated)	-1,78663	3,027989	1,99E-08	1,8E-05
Furin	V-CD8 vs T-CD8 (restimulated)	-1,85112	2,507812	2,52E-08	1,98E-05
Slc16a1	V-CD8 vs T-CD8 (restimulated)	-1,86738	1,535114	7,14E-10	1,2E-06
Fam107b	V-CD8 vs T-CD8 (restimulated)	-1,946344	4,017049	4,06E-09	5,32E-06
Ivns1abp	V-CD8 vs T-CD8 (restimulated)	-2,005129	3,197953	1,92E-12	5,66E-09
Gm49339	V-CD8 vs T-CD8 (restimulated)	-2,09534	2,231179	4,78E-09	5,64E-06
Trbv2	V-CD8 vs T-CD8 (restimulated)	-2,340248	2,343508	2E-14	7,86E-11
Traj5	V-CD8 vs T-CD8 (restimulated)	-2,777148	3,169965	1,93E-14	7,86E-11

Supplementary Table 2: GO and related genes enriched in Vir-CD8 compared to Tum-CD8

ID	Comparison	Description	GeneRatio	p.adjust	geneID
GO:0016032	V-CD8 vs T-CD8 (quiescent)	viral process	10/85	0,000134	Stat1/Vcp/Ptbp1/Bsg/Sp100/Cdc42/Ist1/Ppib/Snx3/Phb
GO:0006753	V-CD8 vs T-CD8 (quiescent)	nucleoside phosphate metabolic p	8/85	0,004975	Vcp/Cnp/Pgk1/Eno1/Gimap7/Myc/Atp5c1/Acly
GO:0045927	V-CD8 vs T-CD8 (quiescent)	positive regulation of growth	7/85	0,004762	Sash3/Dbnl/Serp1/Twf2/Cdc42/Ist1/Ppib
GO:0045862	V-CD8 vs T-CD8 (quiescent)	positive regulation of proteolysis	7/85	0,005346	Stat1/Vcp/Prelid1/Eno1/Myc/Ist1/Ctsd
GO:0007159	V-CD8 vs T-CD8 (quiescent)	leukocyte cell-cell adhesion	7/85	0,005974	Sash3/Skap1/Msn/Prkar1a/Itga4/Dpp4/Itgb2
GO:0050900	V-CD8 vs T-CD8 (quiescent)	leukocyte migration	7/85	0,005974	Msn/Itga4/Dpp4/Bsg/Itgb2/Cdc42/Ppib
GO:0019693	V-CD8 vs T-CD8 (quiescent)	ribose phosphate metabolic proce	7/85	0,006298	Vcp/Pgk1/Eno1/Gimap7/Myc/Atp5c1/Acly
GO:0022613	V-CD8 vs T-CD8 (restimulated)	ribonucleoprotein complex biogen	14/122	5,23E-06	Luc7l/Abce1/Mybbp1a/Eif4b/Usp4/Dkc1/Tsr1/Pwp2/Eif2s3x/Ftsj3/Noc4l/Surf6/Mak16/Celf1
GO:0006913	V-CD8 vs T-CD8 (restimulated)	nucleocytoplasmic transport	12/122	1,11E-05	Ipo5/Hspa9/Abce1/Ranbp2/Nup98/Tnpo3/Cse1l/Thoc1/Ddx5/Xpot/Thoc2/Jak2
GO:0051169	V-CD8 vs T-CD8 (restimulated)	nuclear transport	12/122	1,11E-05	Ipo5/Hspa9/Abce1/Ranbp2/Nup98/Tnpo3/Cse1l/Thoc1/Ddx5/Xpot/Thoc2/Jak2
GO:0008380	V-CD8 vs T-CD8 (restimulated)	RNA splicing	12/122	7,1E-05	Ivns1abp/Luc7l/Usp4/Nup98/Sf3b3/Dhx15/Thoc1/Ddx5/Rbm38/Thoc2/Snrnp70/Celf1
GO:0072594	V-CD8 vs T-CD8 (restimulated)	establishment of protein localizati	11/122	0,000526	Ipo5/Ncoa4/Ranbp2/Dkc1/Nup98/Tnpo3/Cse1l/Ssr3/Ddx5/Rab3gap1/Jak2
GO:0006520	V-CD8 vs T-CD8 (restimulated)	cellular amino acid metabolic proc	9/122	0,000526	Aars/Iars/Shmt1/Ppat/Azin1/Adss/Bcat1/Aldh18a1/Lars

Supplementary Table 3 : List of antibodies used in the study

Reagent	Clone	Source	Cat#	RRID
BUV395 anti-mouse CD8a	53-6.7	BD Biosciences	563786	AB_2732919
BUV737 anti-mouse CD45.1	A20	BD Biosciences	612811	AB_2738850
BV605 anti-mouse CD49a	Ha31/8	BD Biosciences	740519	AB_2740235
FITC anti-mouse IFNγ	XMG1.2	BD Biosciences	562019	AB_395375
PE anti-mouse CD49a	Ha31/8	BD Biosciences	562115	AB_11153117
PE-Cyanine7 anti-mouse CD69	H1.2F3	BD Biosciences	561930	AB_394508
APC anti-mouse CD43 Activation-Associated Glycoform	1B11	Biolegend	121213	AB_528806
APC anti-mouse IL-2	JES6-5H4	Biolegend	503809	AB_315303
BV421 anti-mouse/human Mac-2 (Galectin-3)	M3/38	Biolegend	125416	AB_2566686
BV605 anti-mouse CD45.1	A20	Biolegend	110737	AB_11204076
BV605 anti-mouse/human CD44	IM7	Biolegend	103047	AB_2562451
BV650 anti-mouse TNFα	MP6-XT22	Biolegend	506333	AB_2562450
BV711 anti-mouse CD8a	53-6.7	Biolegend	100748	AB_2562100
FITC anti-rat/mouse-Bcl-2	BCL/10C4	Biolegend	633504	AB_2028394
PE anti-mouse CD43 Activation-Associated Glycoform	1B11	Biolegend	121208	AB_493388
PE-Cyanine7 anti-mouse CD9	MZ3	Biolegend	124815	AB_2783074
PE/Dazzle 594 anti-mouse CD279 (PD-1)	RMP1-30	Biolegend	109116	AB_2566548
CellTrace CFSE Cell Proliferation Kit		Invitrogen	C34554	
CellTrace Violet Cell Proliferation Kit		Invitrogen	C34557	
VioBlue anti-mouse CD44	IM7.8.1	Miltenyi Biotec	130-102-443	
PE anti-mouse TIM-3	215008	R&D Systems	FAB1529P	
Fixable Viability Dye eFluor 780		Thermo Fischer Scientific	65-0865-14	
PE-Cyanine7 anti-mouse CD8a	53-6.7	Thermo Fischer Scientific	25-0081-82	AB_469584
PerCP-eFluor 710 anti-mouse CD49d	R1-2	Thermo Fischer Scientific	46-0492-80	AB_11150051
PerCP-eFluor 710 anti-mouse Ki67	SolA15	Thermo Fischer Scientific	46-5698-82	AB_11040981
